# ATM depletion induces proteasomal degradation of FANCD2 and sensitizes neuroblastoma cells to PARP inhibitors

**DOI:** 10.1186/s12885-023-10772-y

**Published:** 2023-04-05

**Authors:** Sultana Parvin, Jesmin Akter, Hisanori Takenobu, Yutaka Katai, Shunpei Satoh, Ryu Okada, Masayuki Haruta, Kyosuke Mukae, Tomoko Wada, Miki Ohira, Kiyohiro Ando, Takehiko Kamijo

**Affiliations:** 1 Research Institute for Clinical Oncology, Saitama Cancer Center, 818 Komuro, Ina, Saitama 362-0806 Japan; 2 Laboratory of Tumor Molecular Biology, Graduate School of Science and Engineering, Saitama University, Saitama, 338-8570 Japan

**Keywords:** Neuroblastoma, ATM, FANCD2, ATR, RAD51, CRISPR/Cas9, PARP inhibitor

## Abstract

**Background:**

Genomic alterations, including loss of function in chromosome band 11q22-23, are frequently observed in neuroblastoma, which is the most common extracranial childhood tumour. In neuroblastoma, *ATM*, a DNA damage response-associated gene located on 11q22-23, has been linked to tumorigenicity. Genetic changes in *ATM* are heterozygous in most tumours. However, it is unclear how ATM is associated with tumorigenesis and cancer aggressiveness.

**Methods:**

To elucidate its molecular mechanism of action, we established *ATM*-inactivated NGP and CHP-134 neuroblastoma cell lines using CRISPR/Cas9 genome editing. The knock out cells were rigorously characterized by analyzing proliferation, colony forming abilities and responses to PARP inhibitor (Olaparib). Western blot analyses were performed to detect different protein expression related to DNA repair pathway. ShRNA lentiviral vectors were used to knockdown ATM expression in SK-N-AS and SK-N-SH neuroblastoma cell lines. *ATM* knock out cells were stably transfected with FANCD2 expression plasmid to over-expressed the FANCD2. Moreover, knock out cells were treated with proteasome inhibitor MG132 to determine the protein stability of FANCD2. FANCD2, RAD51 and γH2AX protein expressions were determined by Immunofluorescence microscopy.

**Results:**

Haploinsufficient *ATM* resulted in increased proliferation (*p* < 0.01) and cell survival following PARP inhibitor (olaparib) treatment. However, complete *ATM* knockout decreased proliferation (*p* < 0.01) and promoted cell susceptibility to olaparib (*p* < 0.01). Complete loss of ATM suppressed the expression of DNA repair-associated molecules FANCD2 and RAD51 and induced DNA damage in neuroblastoma cells. A marked downregulation of FANCD2 expression was also observed in shRNA-mediated ATM-knockdown neuroblastoma cells. Inhibitor experiments demonstrated that the degradation of FANCD2 was regulated at the protein level through the ubiquitin–proteasome pathway. Reintroduction of FANCD2 expression is sufficient to reverse decreased proliferation mediated by ATM depletion.

**Conclusions:**

Our study revealed the molecular mechanism underlying *ATM* heterozygosity in neuroblastomas and elucidated that *ATM* inactivation enhances the susceptibility of neuroblastoma cells to olaparib treatment. These findings might be useful in the treatment of high-risk NB patients showing *ATM* zygosity and aggressive cancer progression in future.

**Supplementary Information:**

The online version contains supplementary material available at 10.1186/s12885-023-10772-y.

## Background

Neuroblastoma (NB) is the most common childhood extracranial solid tumour of the sympathetic nervous system. In children, NB accounts for 7–10% of cancers and approximately 15% of cancer-related deaths [1, 2]. These tumours exhibit highly heterogeneous clinical behaviour and diverse prognoses. Despite intensive multimodal treatment strategies, tumours in 60–70% of high-risk NB patients show resistant to standard therapy and progress to metastasis [3–5].

The genetic mechanisms underlying NB pathogenesis are not clearly understood. The genetic alterations that are most commonly associated with treatment failure are *MYCN* amplification, ALK activation, *TERT* rearrangement, and mutations in *ATRX* [6–8]. Ploidy status and allelic loss have been associated with cancer aggressiveness and poor prognosis. Other genomic features that represent segmental aberrations include loss of 1p, 11q, and 14q and the allelic gain of 11p and 17q [9, 10]. Deletions in the 11q region have been detected in 11–48% of high-risk NB patients and are associated with poor overall survival, increased relapse probability, and sensitivity to the poly(ADP-ribose) polymerase (PARP) inhibitor (PARPi) olaparib [11]. The 11q region contains important tumour suppressor genes, including ataxia-telangiectasia mutated (*ATM*) on chromosome band 11q22-23 [12]. Intriguingly, 11q heterozygous deletions and *ATM* hemizygous mutations are mutually exclusive in NB tumours [13]. *ATM* knockdown in NB cell lines has been shown to promote tumorigenesis in vitro and in vivo [14]. Functional inactivation of *ATM* has been observed in Ataxia-Telangiectasia (AT) patients who are prone to developing cancer, including thymic lymphoma, breast cancer, and brain cancer [15–17].

ATM and ATR (ATM- and Rad3-related) are members of the phosphatidylinositol 3-kinase-like (PIKK) family of serine/threonine protein kinases. ATM responds to double-strand breaks (DSBs) caused by ionizing radiation (IR) or reactive oxygen species (ROS) and those resulting from physiological processes, such as meiosis, telomere maintenance, and immune system maturation [18]. ATR plays key roles in responding to DNA single-strand breaks (SSBs). More than 700 different proteins are overlapping substrates of ATM and ATR in the DNA damage response (DDR), cell cycle arrest, and transcription. Cell cycle arrest is mediated through the activation of checkpoint kinase 2 (CHK2) and CHK1 by ATM and ATR, respectively [19]. There are several reports on *ATM* gene association and functional mechanisms in the DDR, homologous recombination repair (HRR), and the non-homologous end joining pathway in cancer [20, 21]. However, the mechanisms by which ATM-depleted cells respond to DNA damage and HRR remain unclear.

Fanconi anaemia group D2 protein (FANCD2), which is downstream of ATM, is also associated with the DDR and HRR mechanisms [22]. FANCD2 is a core functional component of the Fanconi anaemia (FA) pathway that participates in HRR by interstrand crosslink (ICL) repair and maintenance of genomic stability [23, 24]. A functional association exists between ATM kinase and FANCD2 in the DDR as ATM phosphorylates FANCD2 at different sites [25]. In recent studies, cells deficient in ATM demonstrated a specific synthetic lethal relationship with FA pathway genes [26]. The aberrant expression of DNA damage responsive genes associated with FA proteins plays a central role in the onset of therapy resistance in many cancers [27, 28]. Despite their frequent use in NBs and other cancers, the therapeutic efficacy of PARPi is limited by cancer cell resistance developed through complex mechanisms involving multiple DNA repair proteins [29].

To investigate the mechanism underlying PARPi-induced cell sensitivity in ATM-deficient human NB cells, we treated clustered regularly interspaced short palindrome repeats (CRISPR)-associated Cas9 nuclease-mediated *ATM*-KO NGP and CHP-134 cells with olaparib. Our study might provide valuable insights related to the treatment of high-risk NB patients showing *ATM* zygosity and aggressive cancer progression.

## Methods

### Cell culture

Human NB cell lines (NGP, CHP134, SK-N-AS and SK-N-SH) were obtained from the American Type Culture Collection (Manassas, VA, USA) and RIKEN Bioresource Cell Bank, Tohoku University Cell Resource Center (Miyagi, Japan). The cells were cultured in RPMI 1640 (Wako, Osaka, Japan) supplemented with 10% heat-inactivated fetal bovine serum (FBS) and 100 μg/mL penicillin/streptomycin (Sigma-Aldrich, St. Louis, MO, USA). Cells were cultured at 37 °C in a 5% CO_2_ incubator. All cell lines that we used in this study were tested and authenticated via STR assay, compared to the database at https://web.expasy.org/cellosaurus. The absence of mycoplasma contamination was confirmed using a Mycoplasma PCR Detection set (Takara Bio, Kusatsu, Shiga, Japan). These analysis was performed within 6 months when this work was completed.

### Chemotherapeutic drugs and compounds

Olaparib (Selleckchem, Houston, TX, USA) and ATM inhibitor (ATMi) KU-55933 (Sigma-Aldrich) were dissolved in dimethyl sulfoxide (DMSO, Sigma-Aldrich). Chemicals were diluted with RPMI 1640 (Wako).

### EditR-inducible CRISPR/Cas9-mediated *ATM* KO of neuroblastoma cell lines

We followed the manufacturer’s protocol to generate haploinsufficient and complete *ATM*-KO NB cell lines. *ATM* wild-type CHP-134 [22] and *ATM* hemizygous NGP [22] cells were transduced with lentiviral particles containing plasmids for the constitutive Cas9 expression (EditR-inducible lentiviral hEF1a-Blast-Cas9 Nuclease Plasmid, #D16010704, Dharmacon, Lafayette, CO, USA). Single guide RNAs (sgRNAs) were designed using the online CRISPR design tool (http://crispr.mit.edu/) to target *ATM*. sgRNAs were designed targeting exons 10 and 11 (Supplementary Fig. S1). Next, we generated cells that stably expressed Cas9 nuclease using EditR lentiviral Cas9 nuclease expression particles. Cas9-expressing cells were selected by blasticidin (Thermo Fisher Scientific). A single Cas9 clone was isolated and cultured for expansion. sgRNA expression particles were then transduced in cells stably expressing Cas9 nuclease. Non-transduced cells were killed by puromycin, and selected single clone was isolated and cultured for expansion. These selected single clones (Cas9 with sgRNA) were defined as control (Ctrl) clone, which are ready to express cas9 and edit *ATM* gene upon doxycycline addition. Freshly prepared doxycycline treatment was applied to induced the expression of cas9 to generate *ATM*-KO cells. We selected two clones with their corresponding control for each sgRNA (for NGP, sgRNA5: Ctrl-3, # 3; Ctrl-4, # 4, and sgRNA6: Ctrl-11, # 11; Ctrl-13, # 13, and CHP-134, sgRNA5: Ctrl-4, # 4; Ctrl-6, # 6, and sgRNA6: Ctrl-1, # 1; Ctrl-4, # 4).

### LentiCRISPRv2-mediated *ATM* KO in NB cell lines

The sgRNAs were cloned into a LentiCRISPRv2 plasmid (#52,961, Addgene, Watertown, MA, USA). We generated *ATM*-KO CHP-134 cells according to a previously described protocol [8].

### Semi-quantitative RT-PCR

Semi-quantitative RT-PCR analyses were conducted as previously described [8, 30, 31]. Total cellular RNA extraction was performed using ISOGEN II (Nippon Gene, Toyama, Japan) or an RNeasy Mini kit (Qiagen, Hilden, Germany). cDNA was synthesized from 2 μg total RNA. The primer sequence is shown in Supplementary Table S1.

### Knockdown of *ATM* by lentiviral gene transduction

pLKO.1-CMV-puromycin-based lentiviral vectors containing five sequence-verified shRNAs targeting human ATM (RefSeqNM_000051) were obtained from the MISSION shRNA library (Sigma-Aldrich) (Supplementary Table S2). The method to prepare and transduce the shRNA lentivirus has been described previously [8, 32]. Two out of the five shRNAs (TRCN0000039948: Sh-1, TRCN0000010299: Sh-5) were selected based on ATM knockdown efficiency.

### FANCD2 over-expression

For stable overexpression of FANCD2, *ATM* KO NGP cells were transfected with pcDNA3.1-flag-FANCD2 with an empty vector (EV), using Lipofectamine LTX and Plus Reagent (Invitrogen), according to the manufacturer’s recommendations. Stably transfected cells were selected in medium containing 80 μg ml^–1^ Zeocine (Invitrogen) and FANCD2 overexpression confirmed by western blotting.

### Western blotting

Western blotting was performed as previously described [8]. The antibody signal was detected using an ECL clarity chemiluminescence kit (Bio-Rad Laboratories, Hercules, CA, USA). Band intensities were quantified using a LAS-4000 luminescent image analyzer (Fujifilm, Tokyo, Japan). The primary and secondary antibodies used in the experiment are shown in Supplementary Table S3.

### Cell proliferation assay

Cells were seeded in 96-well plates at a density of 500 cells per well in a final volume of 100 μL. The culture was maintained at 37 °C with 5% CO_2_. WST-8 labelling solution (10 μL; Cell counting Kit-8; Dojindo, Kumamoto, Japan) was added, and the cells were returned to the incubator for 2 h. The absorbance of the formazan product was detected at 450 nm in a 96-well spectrophotometric plate reader (Infinite 200 PRO; Tecan Trading AG, Männedorf, Switzerland), according to the manufacturer's protocol.

### Colony formation assay

Cells were seeded at a concentration of 500–1000 cells per well in 6-well plates. They were then incubated at 37 °C in 5% CO_2_ to induce colony formation. After 10–15 d, colonies were fixed with 10% (v/v) methanol (Methanol EMSURE ACS, Merck KgAa, Darmstadt, Germany) for 15 min. Cells were stained with 1:20 dilute Giemsa (Giemsa’s azur eosin methylene blue, Merck KgAa) staining solution and deionized water for 30 min, washed twice with deionized water, and air-dried at room temperature.

### Immunofluorescence microscopy

Cells were immunostained as previously described [8, 33]. Nuclei were stained using ProLong Diamond Antifade Mountant with DAPI (Invitrogen, Waltham, MA, USA). Fluorescent images were captured with a BZ-X710 fluorescence microscope (Keyence, Osaka, Japan).

### Statistical analysis

Data are shown as the mean ± standard deviation (SD). Statistically significant differences were determined using a two-tailed paired Student’s *t*-test and one-way ANOVA with Tukey’s multiple comparison test (*p ≤ 0.05, **p ≤ 0.01, and ***p ≤ 0.001; ns, not significant).

## Results

### *ATM* haploinsufficient NB cells exhibit enhanced cell survival

**Fig. 1:**
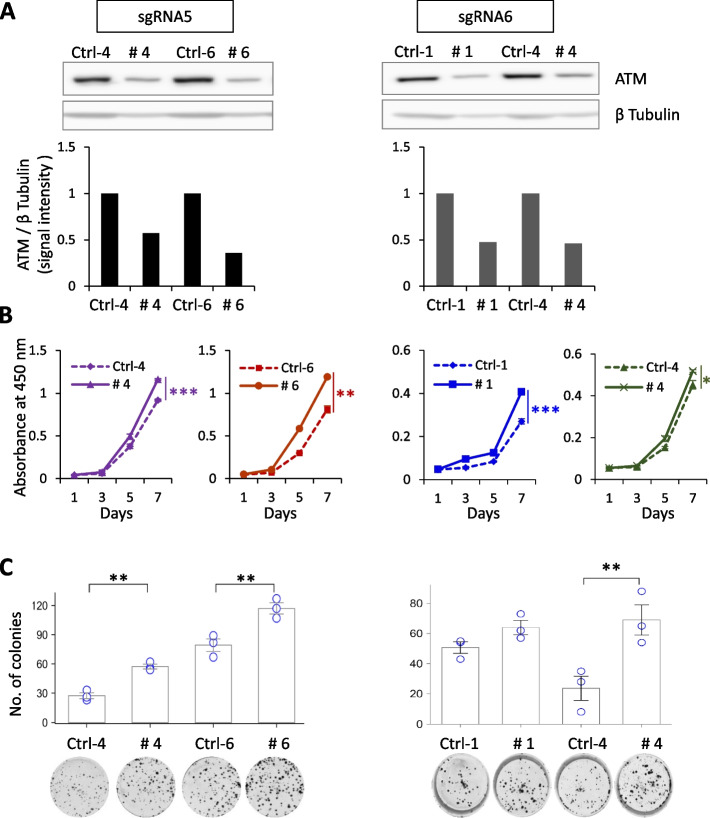
*ATM* haploinsufficiency promotes NB cell proliferation. **A** Western blotting analysis of total ATM. β-Tubulin was used as a loading control. Signal intensities of ATM bands were determined using ImageJ software and normalized using β Tubulin band intensity. **B** Cell proliferation and **C** colony formation assays of *ATM* haploinsufficient CHP-134 cells. Results are presented as the mean ± SD from three independent experiments. *p ≤ 0.05, **p ≤ 0.01, and ***p ≤ 0.001; two-tailed paired Student’s *t*-test. Corresponding uncropped full-length blots are included in Supplementary Materials

CRISPR/Cas9-mediated genome editing represents a powerful approach to determining gene function and the molecular mechanisms underlying complex human diseases. We generated *ATM*-deficient CHP-134 cells using lentiCRISPRv2 and EditR-inducible CRISPR/Cas9 systems. All clones (*n* = 35) showed approximately 50% reduced expression of ATM confirmed by western blotting, suggesting that complete KO clones were not selected (Fig. 1A and Supplementary Fig. S2A, B). The heterozygosity of lenti-CRISPR-mediated CHP-134 *ATM*-KO clones was confirmed by a Sanger sequencing analysis (Supplementary Fig. S2A). Compared to control (Ctrl) cells, all clones showed increased proliferation (Fig. 1B and Supplementary Fig. S2C) and colony numbers (Fig. 1C and Supplementary Fig. S2D).

### ATM-depleted NB cells show decreased proliferation and clonogenic survival

**Fig. 2:**
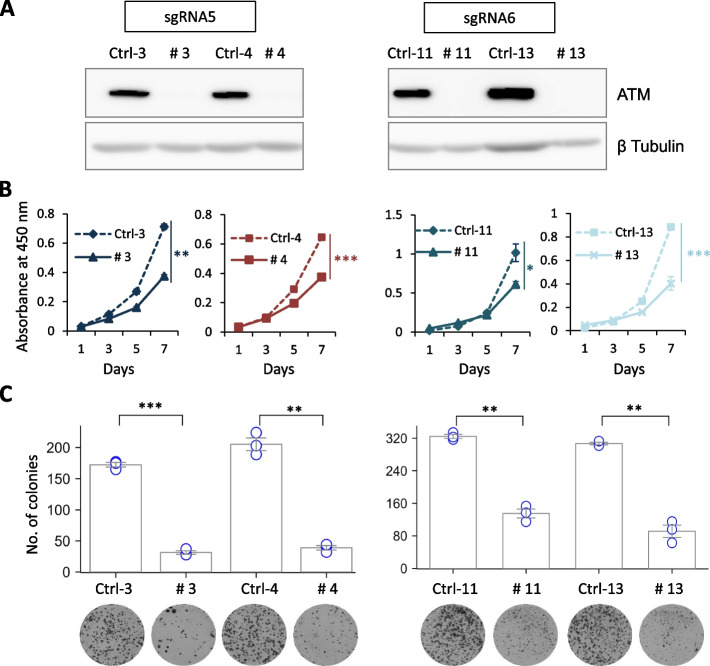
*ATM-*depleted NB cells show decreased proliferation. **A** Western blotting analysis of total ATM. β-Tubulin was used as a loading control. **B** Cell proliferation and **C** colony formation assays of *ATM*-KO NGP cells. Results are presented as mean ± SD from three independent experiments. *p ≤ 0.05, **p ≤ 0.01, and ***p ≤ 0.001; two-tailed paired Student’s *t*-test. Corresponding uncropped full-length blots are included in Supplementary Materials

We generated *ATM*-deficient NGP cells using EditR-inducible CRISPR/Cas9 to avoid biased selection and confirmed the complete loss of ATM by western blot analysis (Fig. 2A). The results showed that ATM loss suppressed NB cell proliferation (*p* < 0.01; Fig. 2B) and colony formation (*p* < 0.01; Fig. 2C) compared to the Ctrl NB cells. This indicates that complete ATM loss inhibits NB cell survival.

### Complete loss of ATM leads to increased endogenous DNA damage through the defective expression of FANCD2 and RAD51

**Fig. 3:**
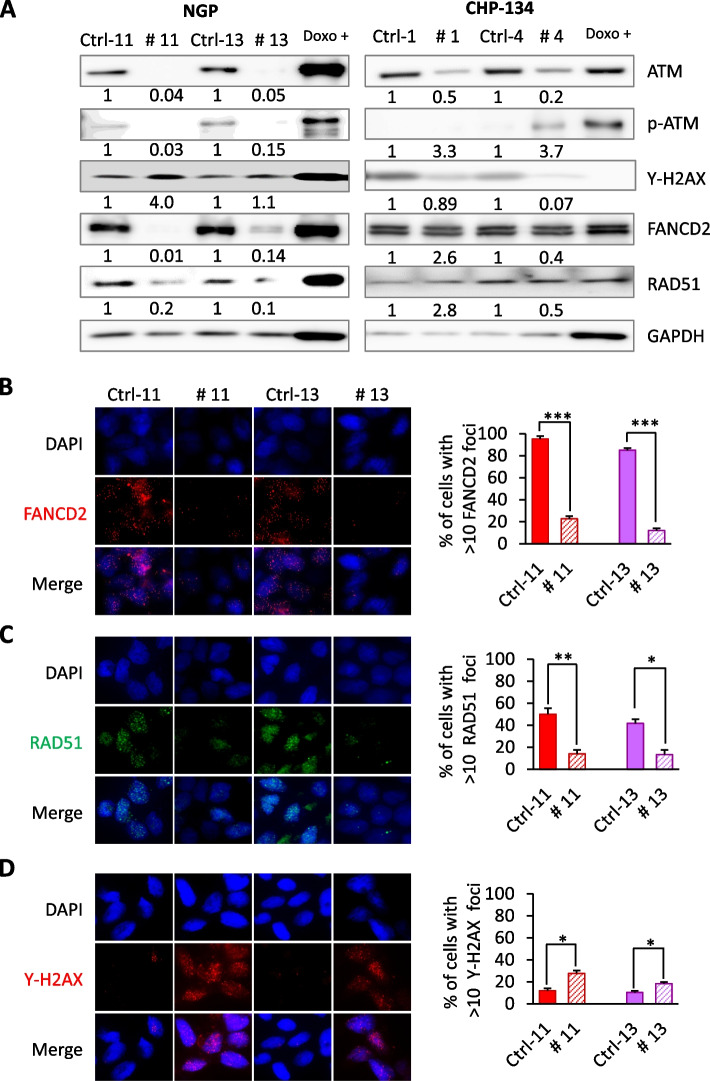
Loss of ATM impairs FANCD2 and RAD51 expression and induces DNA damage. **A** Representative immunoblot images of DDR- and HRR-related molecules in both *ATM*-KO NGP and *ATM* haploinsufficient CHP-134-inducible cells. GAPDH was used as a loading control. Doxorubicin-treated (0.5 μg/mL, 24 h) NGP and CHP-134 cells were used as positive controls. Relative intensities of protein bands were determined using ImageJ software and normalized using loading control band intensity. **B**, **C**, **D** Immunofluorescence and proportion of cells with more than 10 FANCD2 (**B**), RAD51 (**C**), and γH2AX (**D**) foci in *ATM*-deleted NGP cells and their Ctrl counterparts: representative images (left panels) and graphical quantitation of foci (right panels). Nuclear staining with DAPI is indicated in blue. One hundred nuclei were randomly counted. Data are shown as mean ± SD from three independent experiments. *p ≤ 0.05, **p ≤ 0.01, and ***p ≤ 0.001; paired two-tailed Student's *t*-test. Corresponding uncropped full-length blots are included in Supplementary Materials

To study the molecular mechanism of *ATM* inactivation between *ATM* haploinsufficient and complete *ATM*-KO NB cells, we analysed various DDR and HRR-associated proteins (Fig. 3A and Supplementary Fig. S3). The complete loss of ATM in *ATM*-KO NGP cells resulted in increased DSBs, as measured by γH2AX (Fig. 3A and D). As previously stated, *ATM*-edited CHP-134 cells using EditR-inducible CRISPR/Cas9 showed a reduction in ATM of approximately 50% (Fig. 3A). Although heterozygosity was not confirmed by Sanger sequencing due to several integration sites, we designated these cells as “*ATM* haploinsufficient” (data not shown). γH2AX expression were not induced in *ATM* haploinsufficient CHP-134 cells. Previous studies have linked the synthetic lethal relationship of ATM loss with loss of the FA pathway protein, FANCD2 [26]. Consistent with these results [26], we found that FANCD2 levels were reduced in the complete *ATM*-KO NGP cells, leading to decreased cell proliferation. Whereas, in *ATM* haploinsufficient CHP-134 cells FANCD2 levels were unchanged. Simultaneously, ATM loss resulted in a considerable decrease in the expression of RAD51, suggesting defective HRR in *ATM*-KO NGP cells. To further elucidate the impact of complete ATM loss, we immunostained FANCD2, RAD51, and γH2AX in *ATM*-KO NGP cells and their Ctrl counterparts (Fig. 3B, C, and D). We found that the loss of ATM increased the levels of γH2AX foci (Fig. 3D), reflecting endogenous DNA damage. In contrast, the numbers of FANCD2 and RAD51 foci in *ATM*-KO NGP cells were significantly lower than in the corresponding Ctrl cells (Fig. 3B , *p*< 0.001 and Fig. 3C , *p*< 0.01), indicating that the impairment of ATM-mediated HRR function was caused by the complete loss of ATM.

FANCD2 is required for the activation of both ATM-Chk2 and ATR-Chk1 [34]. To test this phenomenon, we compared the status of ATM-Chk2/p53 and ATR-Chk1 activation in the *ATM*-KO NGP and *ATM* haploinsufficient CHP-134 cells by immunoblotting (Supplementary Fig. S3). The complete loss of ATM in NGP cells resulted in the inactivation of both pathways. In contrast, no significant alterations were observed in the *ATM* haploinsufficient CHP-134 cells. These findings suggest that ATM may be responsible for maintaining the FANCD2 function of enhancing ATM-Chk2/p53 and ATR-Chk1 checkpoint activation and suppressing spontaneous DNA damage under normal growth conditions.

### *ATM* knockdown suppresses FANCD2 expression in NB cells

**Fig. 4:**
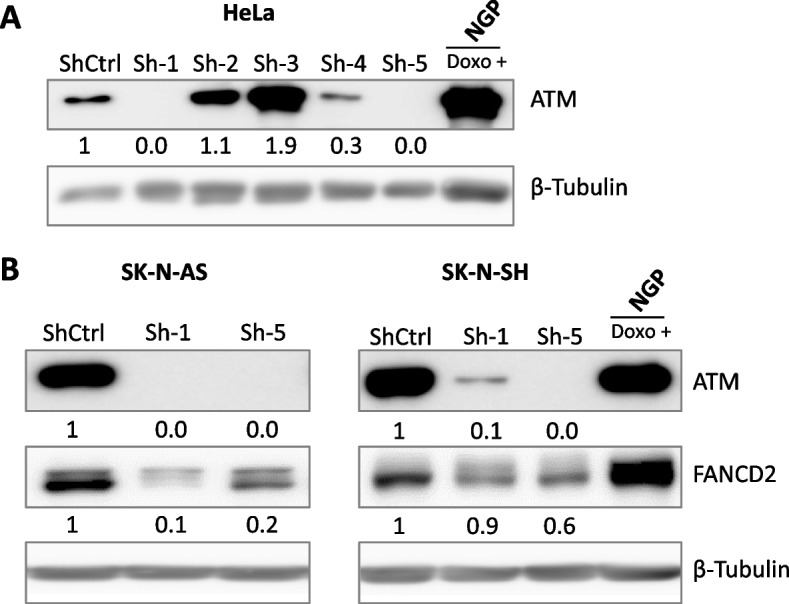
ATM knockdown suppresses FANCD2 expression in NB cells. **A** Western blot analysis showing the silencing efficiency of shRNAs against ATM in HeLa cells. **B** Immunoblotting of ATM and FANCD2 in SK-N-AS and SK-N-SH cells depleted of ATM by shRNAs. NGP cells treated with doxorubicin (0.5 μg/mL, 24 h) were used as a positive control. β-Tubulin served as a loading control. Relative intensities of protein bands were determined using ImageJ software and normalized using loading control band intensity. Corresponding uncropped full-length blots are included in Supplementary Materials

To confirm the relationship between ATM and FANCD2 protein expression, ATM knockdown was performed using lentivirus-mediated shRNA transduction in NB cells. We validated the knockdown efficacy of several ATM shRNAs in HeLa cells and found that shRNA 1 and shRNA 5 efficiently knocked down ATM (Fig. 4A). Compared to shCtrl cells, the expression of FANCD2 was lower in the *ATM-*depleted SK-N-AS and SK-N-SH NB cells (Fig. 4B)*.* These results suggest that the silencing of ATM decreases FANCD2 expression, which is consistent with the results of the inducible CRISPR/Cas9-mediated *ATM*-KO NGP cell experiment.

### ATM loss induces degradation of FANCD2 via the ubiquitin–proteasome pathway

**Fig. 5:**
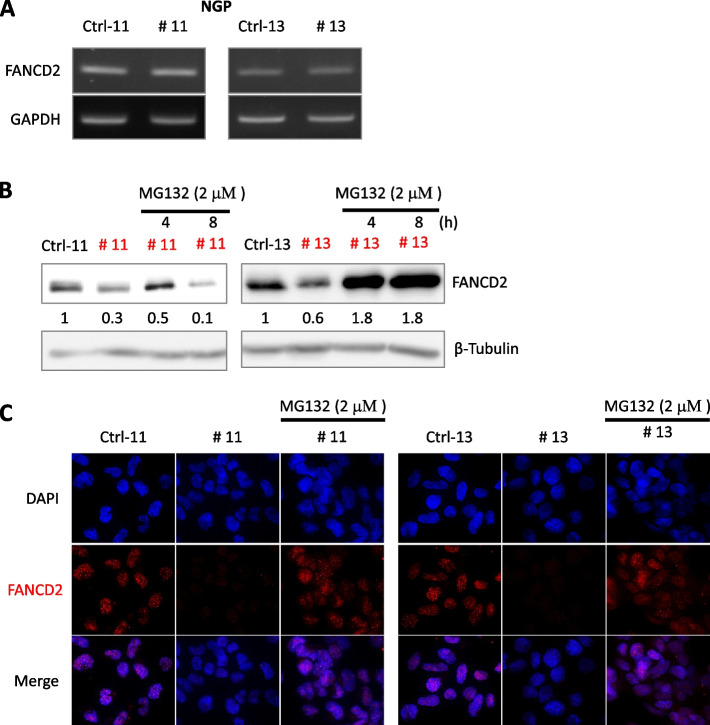
ATM loss triggers FANCD2 degradation in a ubiquitin–proteasome dependent manner. **A** Total RNA was extracted from Ctrl and *ATM*-depleted NGP cells to detect *FANCD2* mRNA by semi-quantitative RT-PCR analyses with GAPDH as an internal control. **B** MG132 inhibits FANCD2 degradation. Proteasome inhibition following MG132 (2 μM) treatment induced FANCD2 accumulation. Whole-cell extracts were analysed via western blotting with anti-FANCD2 and anti–β-Tubulin antibodies. Relative intensities of protein bands were determined using ImageJ software and normalized using loading control band intensity. **C** ATM-depleted NGP cells were treated with 2 µM MG132 for 4 h. FANCD2 was examined using fluorescence microscopy. Cell nuclei were visualized with DAPI staining. Corresponding uncropped full-length blots are included in Supplementary Materials

In the aforementioned experiments, the inhibition of ATM decreased FANCD2 levels. We investigated the underlying mechanism of ATM loss-induced downregulation of FANCD2 in *ATM*-KO NGP cells. We detected *FANCD2* mRNA expression levels by semi-quantitative RT-PCR (Fig. 5A) and found no obvious difference between the *ATM*-KO NGP and Ctrl cells. Thus, ATM may regulate FANCD2 expression by post-translational rather than transcriptional regulation.

The ubiquitin–proteasome system is responsible for the degradation of most intracellular proteins. To investigate the role of proteasomes in the ATM loss-induced downregulation of FANCD2, we treated the *ATM*-KO NGP cells with proteasome inhibitor MG132. The MG132 treatment increased FANCD2 protein levels (Fig. 5B), which was confirmed by immunofluorescence staining with anti-FANCD2 antibody for 4 h (Fig. 5C). Our findings suggest that ATM loss triggers FANCD2 degradation through the ubiquitin–proteasome pathway.

### FANCD2 reintroduction rescue the growth-inhibitory effect of ATM loss in NB cells

**Fig. 6:**
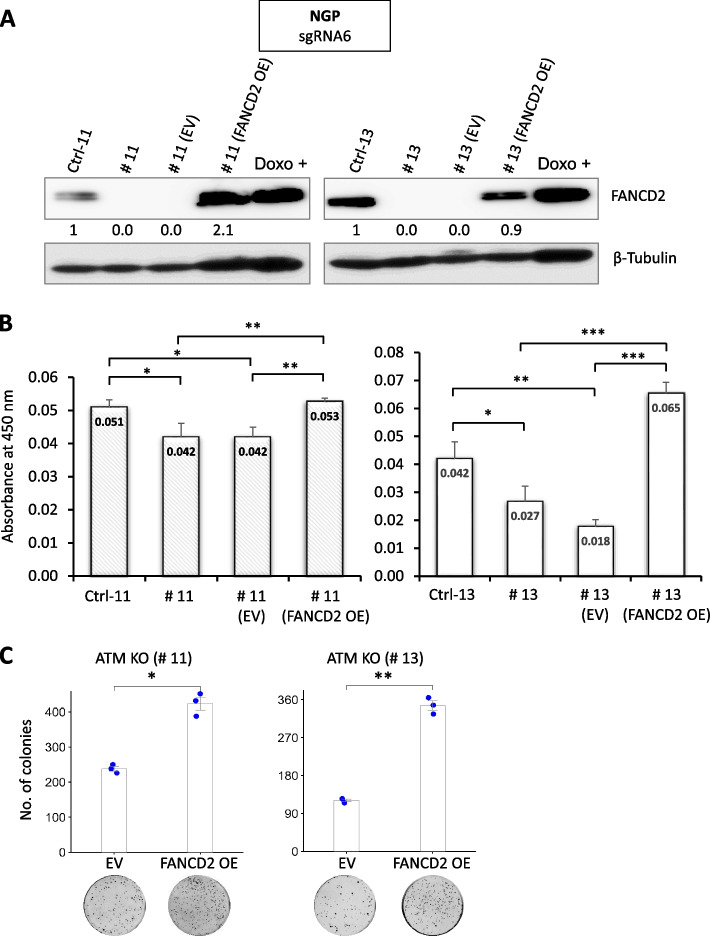
Reintroduction of FANCD2 expression reverse growth suppression mediated by ATM depletion in NB cells. **A** Western blotting analysis of FANCD2. Ctrl and ATM-KO NGP cells (# 11 and # 13) were stably transfected with empty vector (EV) or FANCD2 expression plasmid. β-Tubulin was used as a loading control. Relative intensities of protein bands were determined using ImageJ software and normalized using loading control band intensity. **B** Cell proliferation assay of corresponding Ctrl and *ATM*-KO NGP cells. (# 11 and # 13), stably transfected with empty vector (EV) or FANCD2 expression plasmid. Error bars represent SD from three technical replicates. Statistical analysis via ordinary one-way ANOVA with Tukey’s multiple comparison test (*p ≤ 0.05, **p ≤ 0.01, and ***p ≤ 0.001). **C** Clonogenic assay of *ATM*-KO NGP cells (# 11 and # 13), stably transfected with EV or FANCD2 expression plasmid. Lower panel, representative images for clonogenic formation are shown. The bars represent means with SDs from three experimental replicates. Statistical significance was calculated using two-tailed paired Student’s t-test. *p < 0.05 and **p < 0.01. Corresponding uncropped full-length blots are included in Supplementary Materials

Since ATM deficient cells were sensitive due to loss of FANCD2 expression (Figs. 2 and 3A), we asked whether the reintroduction of FANCD2 into *ATM*-KO NGP cells could rescue the growth-inhibitory effect of ATM loss. Therefore, we stably overexpressed FANCD2 in *ATM*-KO NGP cells with an empty vector (Fig. 6A). Reintroduction of FANCD2 led to an increase proliferation rate of *ATM*-KO NGP cells compared with the proliferation rate of empty vector–containing *ATM*-KO cells (*p* < 0.001; Fig. 6B). Furthermore, in the flat colony formation assay, the colony forming ability of FANCD2-overexpressing *ATM*-KO NGP cells were significantly induced compared to the empty vector transfected cells (*p* < 0.05, *p* < 0.01; Fig. 6C). This indicates that FANCD2 reintroduction in *ATM*-KO NGP cells restored its growth suppressor activity.

### ATM deficiency enhances the inhibitory effects of PARPi in NB cells

**Fig. 7:**
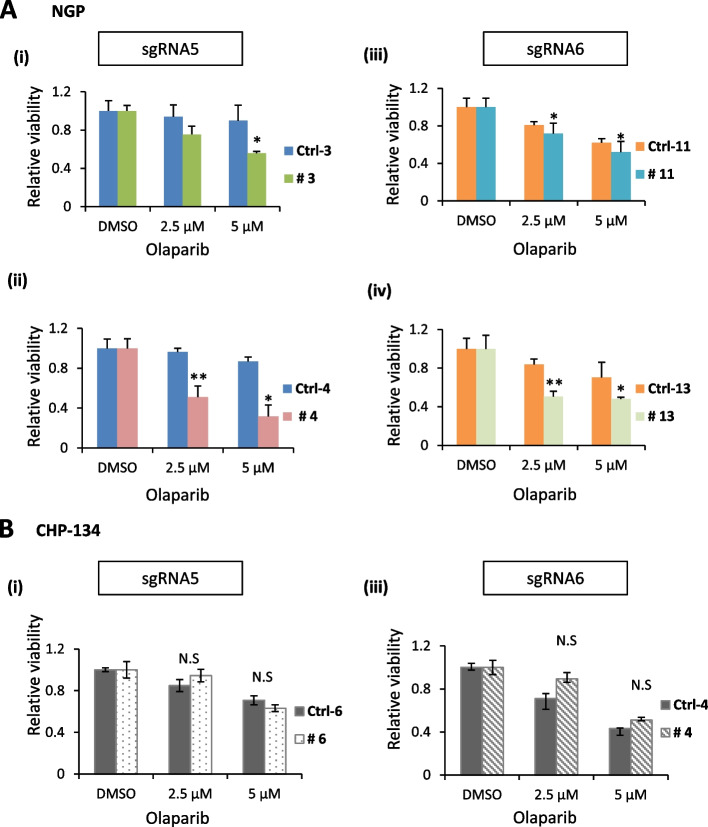
ATM deficiency enhances inhibitory effect of PARPi in NB cells. Olaparib-treated CRISPR-Ctrl and -ATM cells were evaluated for viability. Cells were plated at 3000 cells/well/100 µL. WST-8 assays were performed 72 h after olaparib treatment. DMSO was used as a negative control. **A**, **B ***ATM*-KO NGP cells and *ATM* haploinsufficient CHP-134 cells against olaparib viability, respectively. Data are shown as mean ± SD from three independent repeats. Statistical significance was calculated using the two-tailed paired Student’s t-test, where *p ≤ 0.05, **p ≤ 0.01, and N.S, not significant

Olaparib is a widely used PARPi in the treatment of NB and other cancers [16, 17]. Functionally defective DDRs are reportedly regulated by ATM in many NB-derived cell lines [13]. In addition, ATM has been associated with HRR, and ATM-deleted mantle cell lymphoma showed increased sensitivity to PARPi [35]. We therefore investigated the impact of PARP inhibition on NB cell susceptibility. We treated CRISPR/Cas9-mediated *ATM-*KO NGP cells with olaparib in a dose-dependent manner and found that *ATM*^*−*^KO clones showed high sensitivity to treatment at 2.5 and 5.0 μM (Fig. 7A). Conversely, *ATM* haploinsufficient and *ATM* heterozygous NB cells showed resistant phenotypes (Fig. 7B and Supplementary Fig. S2E). Furthermore, the combination treatment of PARPi Olaparib and ATMi KU-55933 significantly decreased the cell survival in *ATM* wild type CHP-134 Ctrl (Ctrl-4) cells. Even in the *ATM* haploinsufficient CHP-134 cells (# 4) that were resistant to PARPi, the combination of PARPi and ATMi can suppress the cell proliferation (Supplementary Fig. S4).

## Discussion

Modern cancer research is faced with monumental challenges including adverse side effects and resistance to chemically or molecularly targeted therapies caused by unknown mechanisms. Molecular targeted therapies share many common features, such as alterations of the drug target (e.g., genetic aberrations), inactivation of pro-survival pathways, and induction of cell death [4]. Mutations, including allelic deletions in the *ATM* tumour suppressor gene, are common in all cancers [36, 37]. These mutations can cause neurodegenerative diseases and cancer-predisposition syndrome. They may also affect cell sensitivity to various clinical DNA damaging agents, such as topotecan and olaparib [38]. It has also been hypothesized that haploinsufficient *ATM* causes the removal of DNA repair genes and promotes carcinogenesis in leukaemia cells [12]. In the present study, we established *ATM*-KO NB cells for the first time using lentiviral-mediated stable and inducible CRISPR/Cas9 genome editing (Supplementary Fig. S1). We investigated the tumorigenic function of *ATM* haploinsufficient and heterozygous CHP-134 and ATM-depleted NGP NB cell lines, respectively, in proliferation and colony formation assays. *ATM* haploinsufficiency and heterozygous deletions significantly enhanced cell viability as confirmed by WST-8 and colony forming assays (Fig. 2A–C and Supplementary Fig. S2A–D). Similarly, a previous study reported that *ATM* knockdown enhanced tumorigenic functions in SK-N-SH, CLB-GA, and GI-ME-N NB cell lines by potentially inhibiting DNA repair [12]. We found that complete *ATM* depletion significantly suppressed NB cell proliferation and colony formation (Fig. 2A–C) and induced hypersensitivity to olaparib in NGP cells (Fig. 7Ai–iv). Conversely, olaparib treatment in *ATM* haploinsufficient and heterozygous CHP-134 cells was ineffective (Fig. 7Bi–ii and Supplementary Fig. S2E).

Since *ATM* is a DDR gene and functions through the phosphorylation of HRR-associated genes, namely *ATR*, *RAD51*, *FANCD2*, *RPA2*, and *BRCA1/2* among others [39–43], we investigated the expression of various HRR-associated genes and γH2AX in *ATM*-KO NGP and *ATM* haploinsufficient CHP-134 cells. We observed that the protein levels of FANCD2, RAD51, and ATR, which promote alternative end-joining, DNA damage repair, and cancer cell survival [44, 45], were downregulated. In contrast, p21 and γH2AX levels increased in the *ATM*-deficient NB cells (Fig. 3A and Supplementary Fig. S3). No significant changes were observed in the expression of FANCD2, ATR, P-ATR, and p53 in the *ATM* haploinsufficient NB cells, however. This is consistent with previous findings in which ATM inhibition profoundly decreased RAD51 foci formation, increased DNA damage or γH2AX foci formation, and impaired HRR through the downregulation of RAD51 in human glioblastoma, lung, and cervical carcinoma cells [46, 47]. Another study found that ATM inhibition or loss of FANCD2 conferred a reduction in HRR and RAD51 foci formation in lung cancer [26], which is consistent with our finding that complete ATM loss in NGP cells impaired HRR through the downregulation of FANCD2 and RAD51 expression.

Since ATM loss led to decreased FANCD2 expression at the protein level but not at the mRNA level (Fig. 5A and B), we investigated the role of proteasomes in the ATM loss-induced downregulation of FANCD2. Proteasome inhibitor MG132 treatment in *ATM*-KO NGP cells upregulated the expression of FANCD2 at the protein level, as confirmed by immunofluorescence staining (Fig. 5B and C). This indicated that ATM loss triggers FANCD2 degradation through the ubiquitin–proteasome pathway. Moreover, reintroduction of FANCD2 rescue the growth-inhibitory effect of ATM loss in NB cells. Our study also supports the findings by a another research group who reported enhanced sensitivity to PARP inhibition in NB cells following 11q deletion [13, 48], though the SK-N-AS cell line in their study showed resistance [48]. Along with 11q deletion, *ATM* zygosity status is a critical determinant of sensitivity to PARPi in NB cells. In the present study, we found that 11q-deleted parental NGP cells with an *ATM* hemizygous status showed enhanced survival to PARPi compared to cells with complete ATM loss. Our results were further consistent in the CHP-134 NB cell line.

## Conclusions

We demonstrated the clinical relevance and key molecular mechanisms of ATM inactivation in NB clones (Supplementary Fig. S5). CRISPR/Cas9-mediated complete *ATM* depletion suppressed cell survival and enhanced susceptibility to PARPi in NB cells through the impairment of ATM-mediated HRR. Our findings will be significant to researchers and physicians in the field of precision medicine and suggest a novel therapeutic component for treating high-risk NB patients showing *ATM* zygosity and aggressive cancer progression.

## Supplementary Information

**Additional file 1: Supplementary Figure S1.** Generation of CRISPR/Cas9-mediated ATM-depleted NB cells. **Supplementary Figure S2.** Phenotypic analysis of ATM heterozygous CHP-134 NB cells. **Supplementary Figure S3.** ATM is required for both ATM/Chk2/p53 and ATR/Chk1 pathway activation. **Supplementary Figure S4.** Combination treatment (ATMi KU-55933 + PARPi Olaparib), reversed resistance to PARPi in ATM haploinsufficient CHP-134 cells. **Supplementary Figure S5.** Loss of function in ATM suppresses tumorigenicity and sensitizes NB cells to PARPi. **Supplementary Table S1.** List of primer sequences used in this study. **Supplementary Table S2.** Targeting sequences of shRNAs against human ATM used in this study. **Supplementary Table S3.** List of antibodies used in this study.

## Data Availability

All data needed to evaluate the conclusion in the paper are present in the paper and/or the supplementary materials.

## Abbreviations

ATM: Ataxia-telangiectasia mutated
CRISPR: Clustered regularly interspaced short palindromic repeats
DDR: DNA damage response
FANCD2: Fanconi anaemia group D2 protein
FBS: Foetal bovine serum
HRR: Homologous recombination repair
KO: Knockout
NB: Neuroblastoma
PARP: Poly (ADP-ribose) polymerase
sgRNA: Single guide RNA
shRNA: Small hairpin RNA
