# Supplementary material for: ATM depletion induces proteasomal degradation of FANCD2 and sensitizes neuroblastoma cells to PARP inhibitors
Source: BMC Cancer. 2023 Apr 5;23:313. doi: 10.1186/s12885-023-10772-y (PMC10077671; doi:10.1186/s12885-023-10772-y)
Supplement: Supplementary file 1 — Additional file 1: Supplementary Figure S1. Generation of CRISPR/Cas9-mediated ATM-depleted NB cells. Supplementary Figure S2. Phenotypic analysis of ATM heterozygous CHP-134 NB cells. Supplementary Figure S3. ATM is required for both ATM/Chk2/p53 and ATR/Chk1 pathway activation. Supplementary Figure S4. Combination treatment (ATMi KU-55933 + PARPi Olaparib), reversed resistance to PARPi in ATM haploinsufficient CHP-134 cells. Supplementary Figure S5. Loss of function in ATM suppresses tumorigenicity and sensitizes NB cells to PARPi. Supplementary Table S1. List of primer sequences used in this study. Supplementary Table S2. Targeting sequences of shRNAs against human ATM used in this study. Supplementary Table S3. List of antibodies used in this study. [file 12885_2023_10772_MOESM1_ESM.pdf]

Supplementary material

Title page

ATM depletion induces proteasomal degradation of FANCD2 and sensitizes neuroblastoma cells to PARP inhibitors

Sultana Parvin[1,2,†], Jesmin Akter[1,†], Hisanori Takenobu[1], Yutaka Katai[1], Shunpei Satoh[1], Ryu Okada[1,2], Masayuki Haruta[1], Kyosuke Mukae[1], Tomoko Wada[1], Miki Ohira[1], Kiyohiro Ando[1] and Takehiko Kamijo[1,2,*]

[1]Research Institute for Clinical Oncology, Saitama Cancer Center, 818 Komuro, Ina, Saitama, 362-0806, Japan

[2]Laboratory of Tumor Molecular Biology, Graduate School of Science and Engineering, Saitama University, Saitama, 338-8570, Japan

*To whom correspondence should be addressed: Tel: 7630 81-48-722-1111; Email: tkamijo@saitama-pho.jp

[†] These authors contributed equally to this work.

Supplementary Figure

Supplementary Figure S1. Generation of CRISPR/Cas9-mediated *ATM*-depleted NB cells. We generated *ATM*-knockout and *ATM* haploinsufficient NB cells using lentiCRISPRv2 and EditR-inducible CRISPR/Cas9 genome editing. (**A**) We selected NB cell lines based on the status of major mutant genes associated with NB pathogenesis and aggressiveness, including MYCN, *P53* mutation, 11q deletion, and zygosity status of ATM. (**B, C**) We designed sgRNA-5 and sgRNA-6 targeting ATM at exon 10 and 11, respectively, at the 11q loci of chromosome 11.

A

**Cell line name**	**Male/ Female**	***MYCN* status**	***ATM* deletion**	***P53* mutation**	**11q deletion**
NGP	Male	Amplified	Hemizygous	WT	Loss q22.1-qter
CHP-134	Male	Amplified	WT	WT	No
SK-N-SH	Female	Single	WT	F1174L	No
SK-N-AS	Female	Single	Hemizygous	WT	Yes

B

List of single guide RNA target sequences

**sgRNA number**	**sgRNA target sequence**
ATM sgRNA # 5	ATATGTGTTACGATGCCTTA
ATM sgRNA # 6	CTGACCACCAGTATAGTTCC

sgRNA, single guide RNA

C

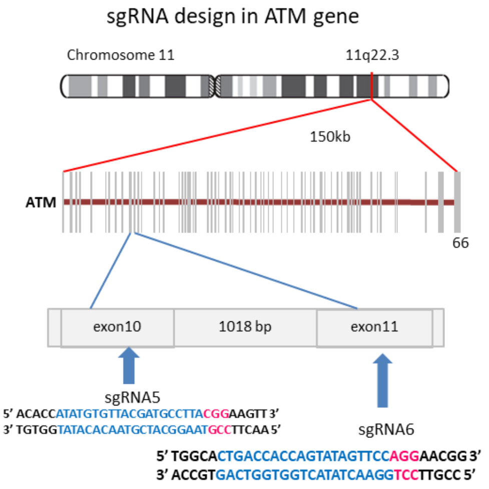

Supplementary Figure S2. Phenotypic analysis of *ATM* heterozygous CHP-134 NB cells. (**A**). *ATM* heterozygous clones were confirmed by sequencing. (**B**) Western blot analysis for ATM; β-Tubulin was used as a loading control. ATM/β-Tubulin protein intensity was assessed with a Cas9 control. (**C**) Cell proliferation and (**D**) colony formation assays of *ATM* heterozygous CHP-134 clones. (**E**) *ATM* heterozygous KO clones treated with PARP inhibitor olaparib. Data are shown as mean ± SD from three independent experiments. *$p \leq 0.05$, **$p \leq 0.01$, and ***$p \leq 0.001$; paired two-tailed Student's *t*-test.

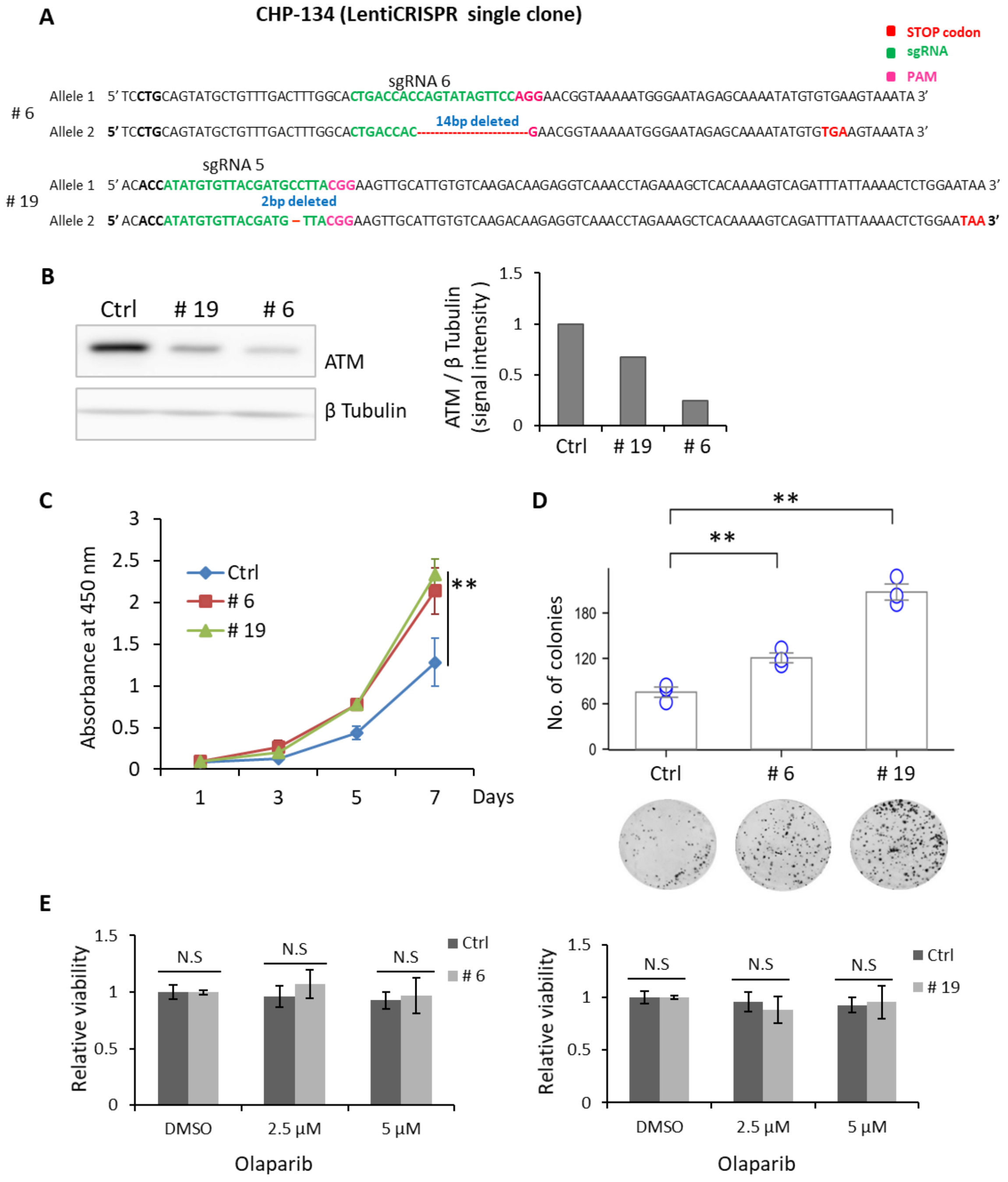

Supplementary Figure S3. ATM is required for both ATM/Chk2/p53 and ATR/Chk1 pathway activation. Representative western blot analysis of the indicated proteins in cell lysates prepared from Ctrl, *ATM*-KO NGP, and *ATM* haploinsufficient CHP-134 cells. β-Tubulin was used as a loading control. Doxorubicin-treated (0.5 μg/mL, 24 h) NGP and CHP-134 cells were used as positive controls.

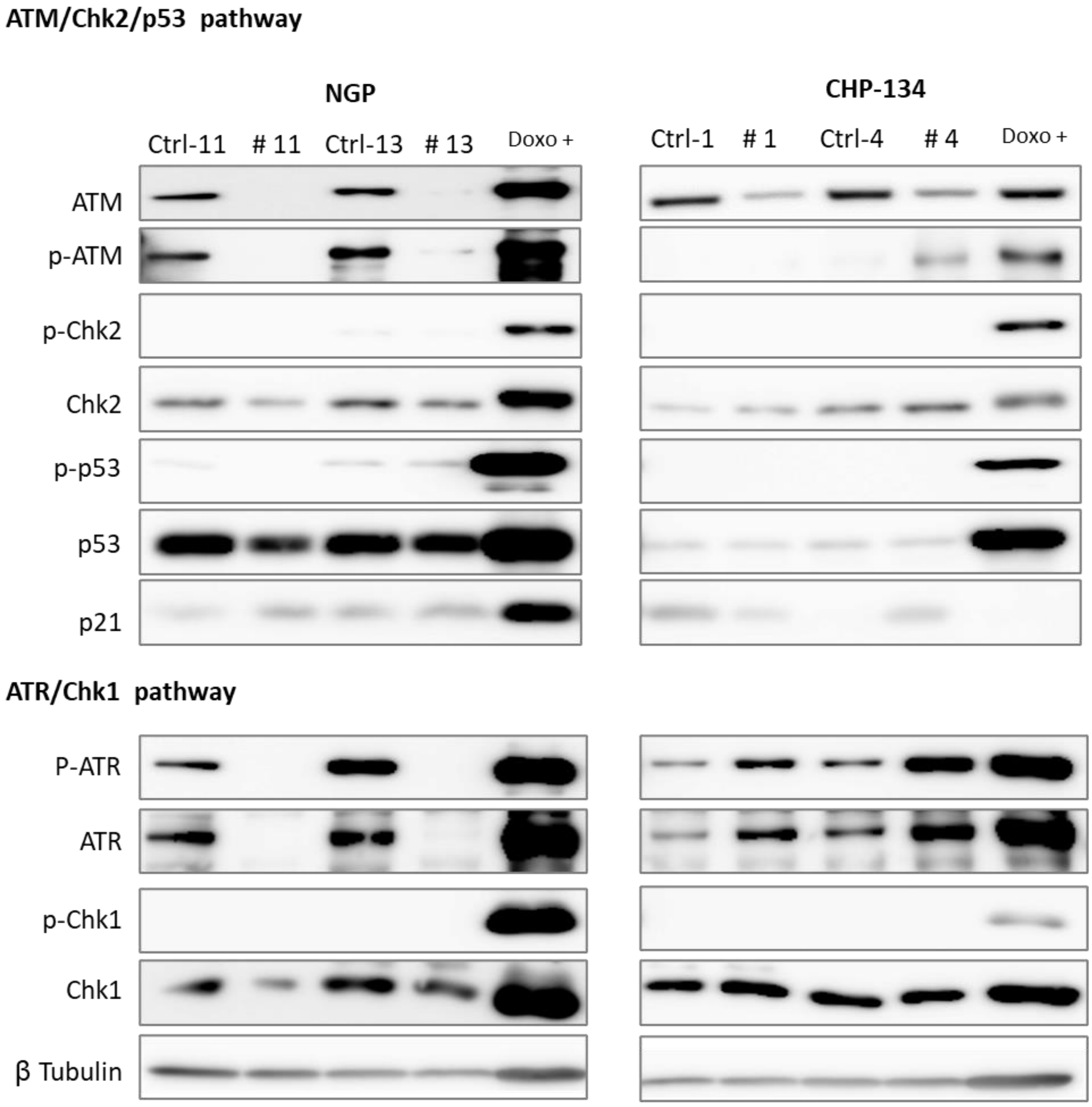

Supplementary Figure S4.

Combination treatment (ATMi KU-55933 + PARPi Olaparib), reversed resistance to PARPi in *ATM* haploinsufficient CHP-134 cells. *ATM* haploinsufficient CHP-134 cells and Ctrl counterparts were plated at 3000 cells/well/100 µL. WST-8 assays were performed 72 h after ATMi KU-55933, PARPi Olaparib or combination treatment. DMSO was used as a negative control. N.S; not significant, *$p \leq 0.05$, **$p \leq 0.01$, and ***$p \leq 0.001$; two-tailed Student's *t*-test.

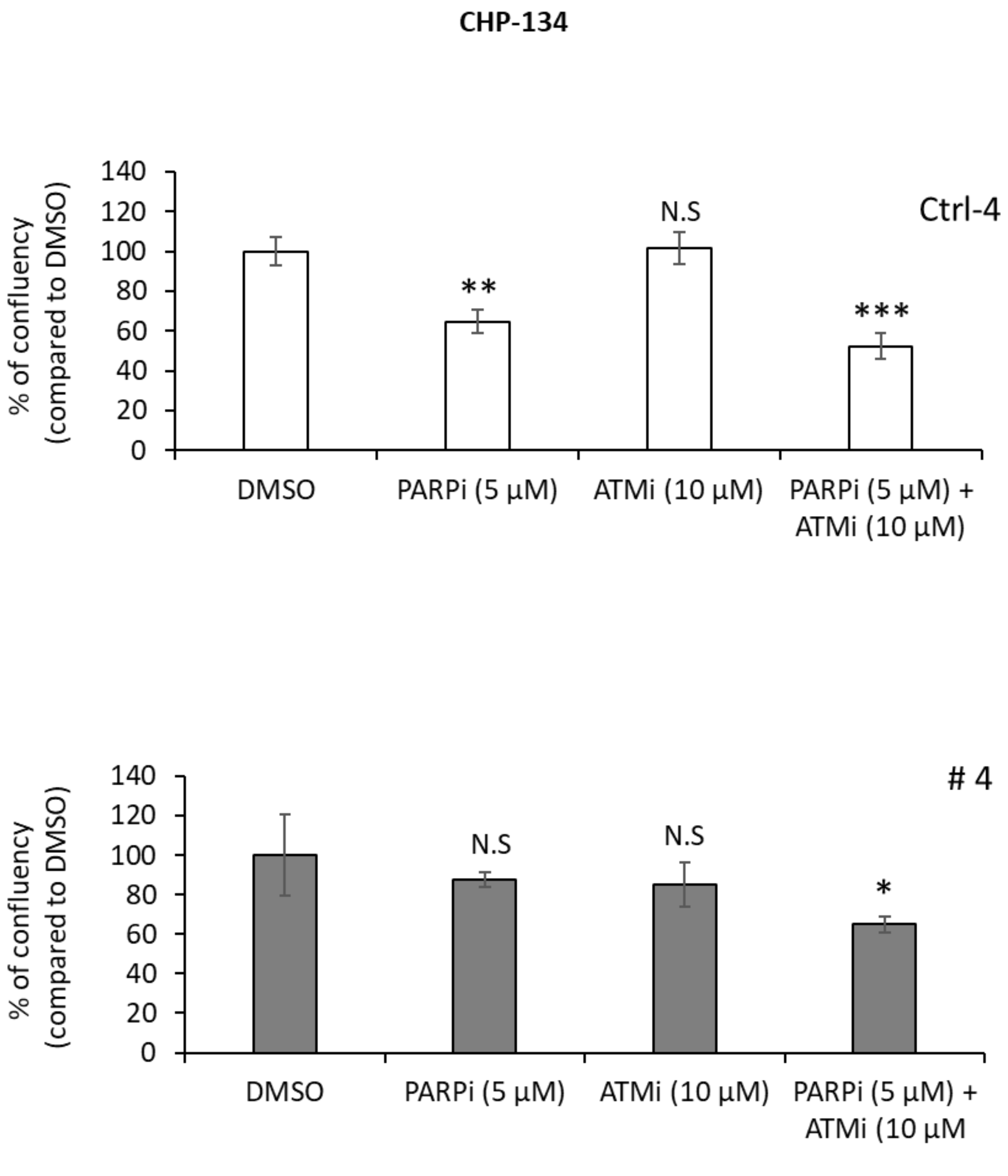

Supplementary Figure S5.

Loss of function in ATM suppresses tumorigenicity and sensitizes NB cells to PARPi. CRISPR/Cas9-mediated *ATM* KO in NB cells suppressed tumorigenicity and enhanced susceptibility to olaparib treatment through the inactivation of the FA pathway. Haploinsufficient NB cells showed resistance against PARPi through an unknown mechanism.

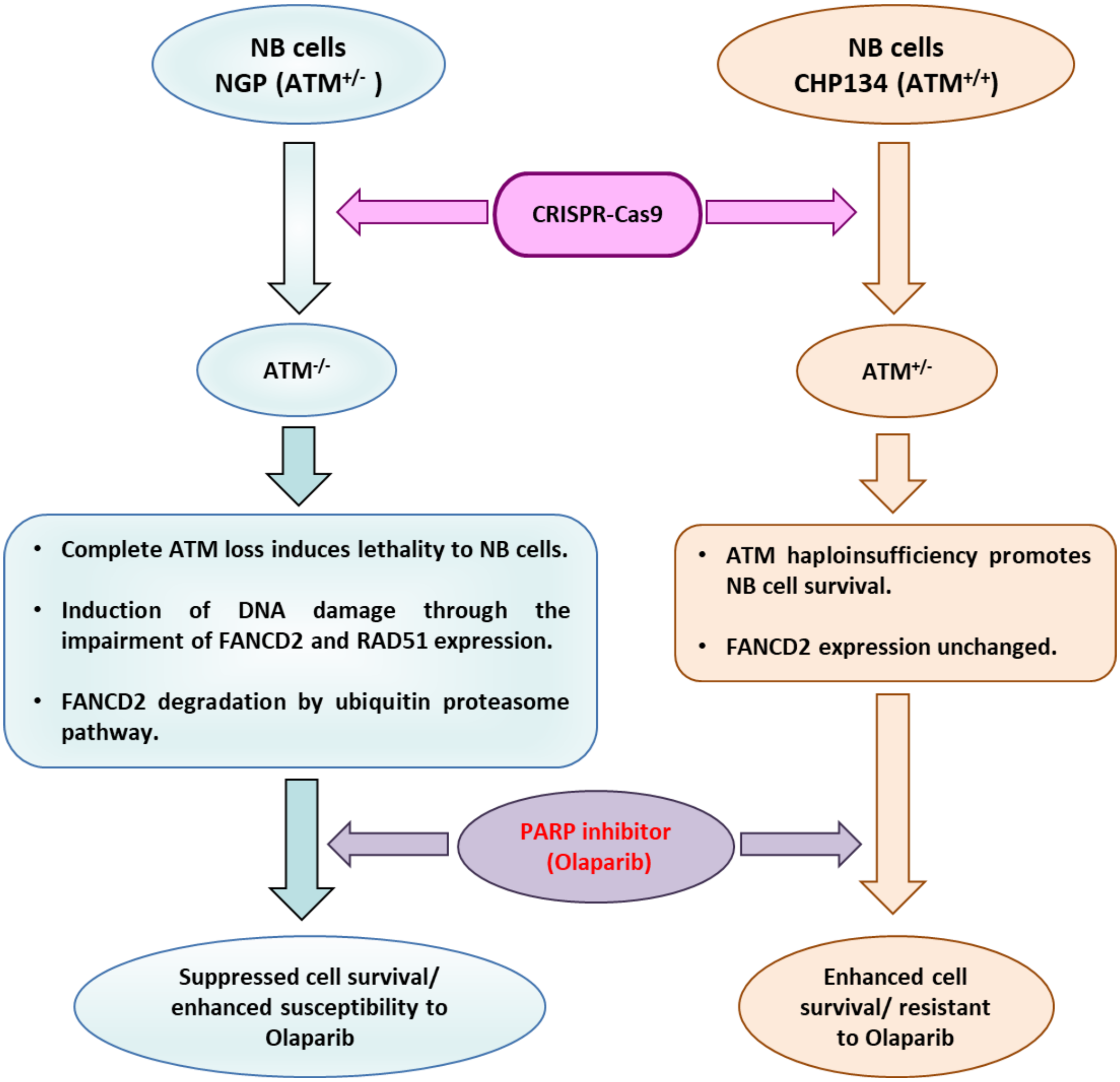

Supplementary Table S1. List of primer sequences used in this study.

Gene name	Forward primer sequences (5'→3')	Reverse primer sequences (5'→3')
Rt-PCR primer		
FANCD2	ACATACCTCGACTCATTGTCAGT	TCGGAGGCTTGAAAGGACATC
GAPDH	ACCACAGTCCATGCCATCAC	TCCACCACCCTGTTGCTGTA

Supplementary Table S2. Targeting sequences of shRNAs against human *ATM* used in this study.

shRNA	Clone ID (Sigma)	Clone Name	Target sequence
Sh-1	TRCN0000039948	NM_000051	CCTTTCATTCAGCCTTTAGAA
Sh-2	TRCN0000039951	NM_000051	GCCTCCAATTCTTCACAGTAA
Sh-3	TRCN0000038658	NM_000051	CGTGTCTTAATGAGACTACAA
Sh-4	TRCN0000194861	NM_000051	CCAAGGTCTATGATATGCTTA
Sh-5	TRCN0000010299	NM_000051	TGATGGTCTTAAGGAACATCT

Supplementary Table S3: List of antibodies used in this study.

Primary antibodies	Merchant, Catalog number	Species, type	Application
ATM	Santa Cruz (2C1): sc-23921	Mouse, Monoclonal	WB (1:1000)
Phospho–ATM Ser 1981	Millipore 10H11.E12, 05-740	Mouse, Monoclonal	WB (1:1000)
Phospho-γH2AX Ser 139	Millipore (JBW301), 05-636	Mouse, Monoclonal	WB (1:4000)
Phospho-Histone H2AX Ser 139	Cell signaling, 2577	Rabbit	IF (1:100)
ATR	Cell signaling, 2790	Rabbit, Polyclonal	WB (1:1000)
Phospho-ATR Ser 428	Cell signaling, 2853	Rabbit, Polyclonal	WB (1:1000)
Phospho–Chk1 Ser 345	Cell signaling, 13303	Rabbit, Polyclonal	WB (1:1000)
Chk1	Santa Cruz (G4): sc-8408	Mouse, Monoclonal	WB (1:1000)
Phospho–Chk2 Thr 68	Cell signaling (C13C1), 2197	Rabbit, Monoclonal	WB (1:1000)
Chk2	Merck (Clone 7), 05-649	Mouse, Monoclonal	WB (1:1000)
Phospho–p53 Ser 15	Cell signaling, 9284	Rabbit, Polyclonal	WB (1:1000)
p53	Santa Cruz (DO-1): sc-126	Mouse, Monoclonal	WB (1:1000)
p21	Santa Cruz (187): sc-817	Mouse, Monoclonal	WB (1:1000)
FANCD2	Novus Biologics, NB100-182	Rabbit, Polyclonal	WB (1:3000); IF (1:200)
RAD51	Novus Biologics, NB100-148	Mouse, Monoclonal	WB (1:1000); IF (1:100)
GAPDH	Wako, 5A12	Mouse, Monoclonal	WB (1:2000)
β-Tubulin	Wako, 10G10	Mouse, Monoclonal	WB (1:2000)
**Secondary antibodies**	**Merchant, Catalog number**	**Species, type**	**Application**
Anti-Rabbit IgG (H+L) HRP	MBL, 458	Rabbit, Polyclonal	WB (1:6000)
Anti-Mouse IgG (H+L) HRP	MBL, 330	Mouse, Polyclonal	WB (1:6000)
Alexa fluor 594 F(ab')2-goat anti-rabbit IgG (H+L)	Molecular probe, A11072	Rabbit, Polyclonal	IF (1:1000)
Alexa fluor 488 F(ab')2-goat anti-mouse IgG (H+L)	Molecular probe, A11017	Mouse, Polyclonal	IF (1:1000)

Full-length blots of Figure 1

Figure 1A

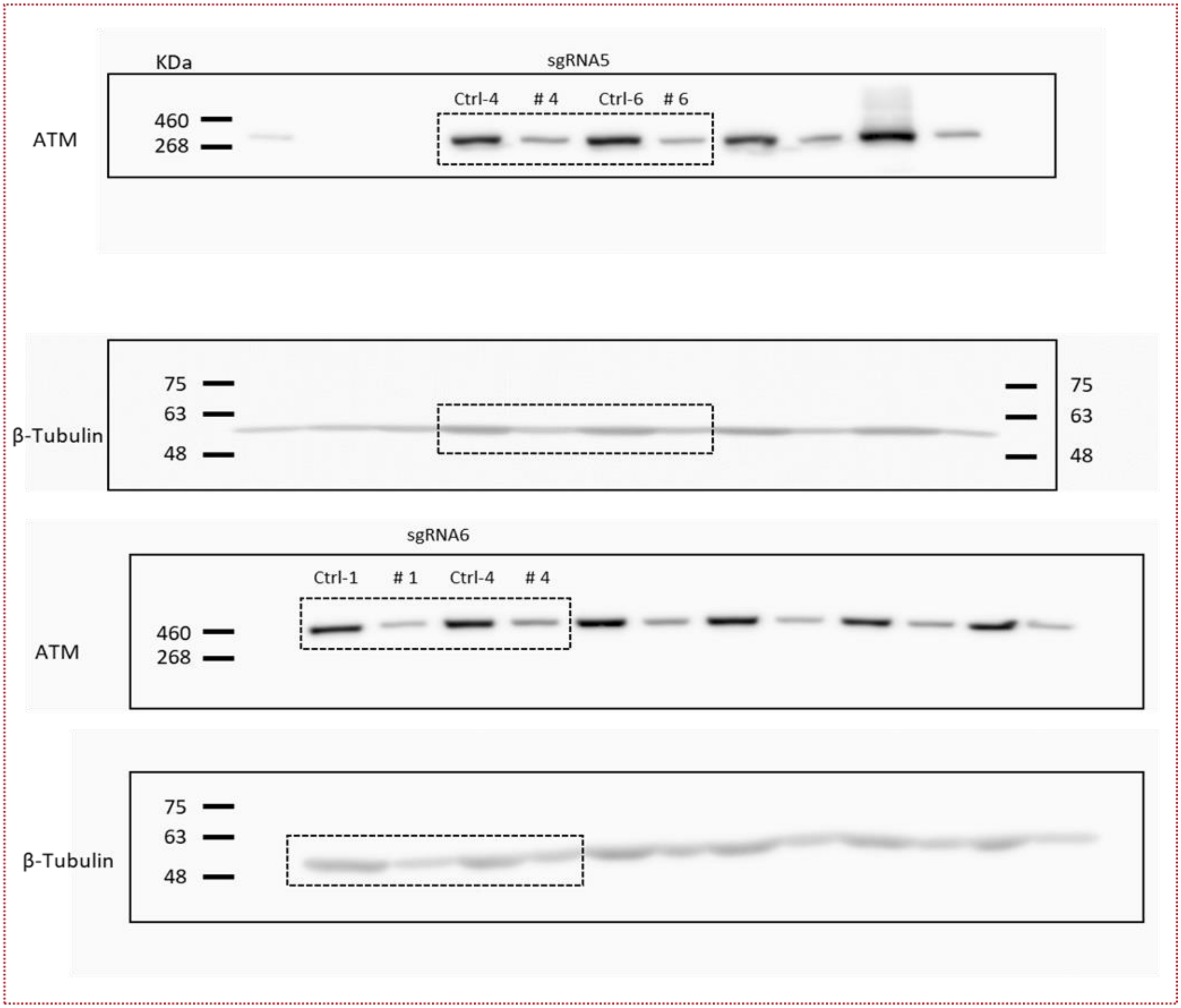

Full images of western blots shown in Figure 1A. Original full-length membrane was divided in to several parts to hybridization with different antibodies. Black block line indicates the edges of the membrane after cut. Black dotted lines indicate the cropping locations.

Full-length blots of Figure 2

Figure 2A

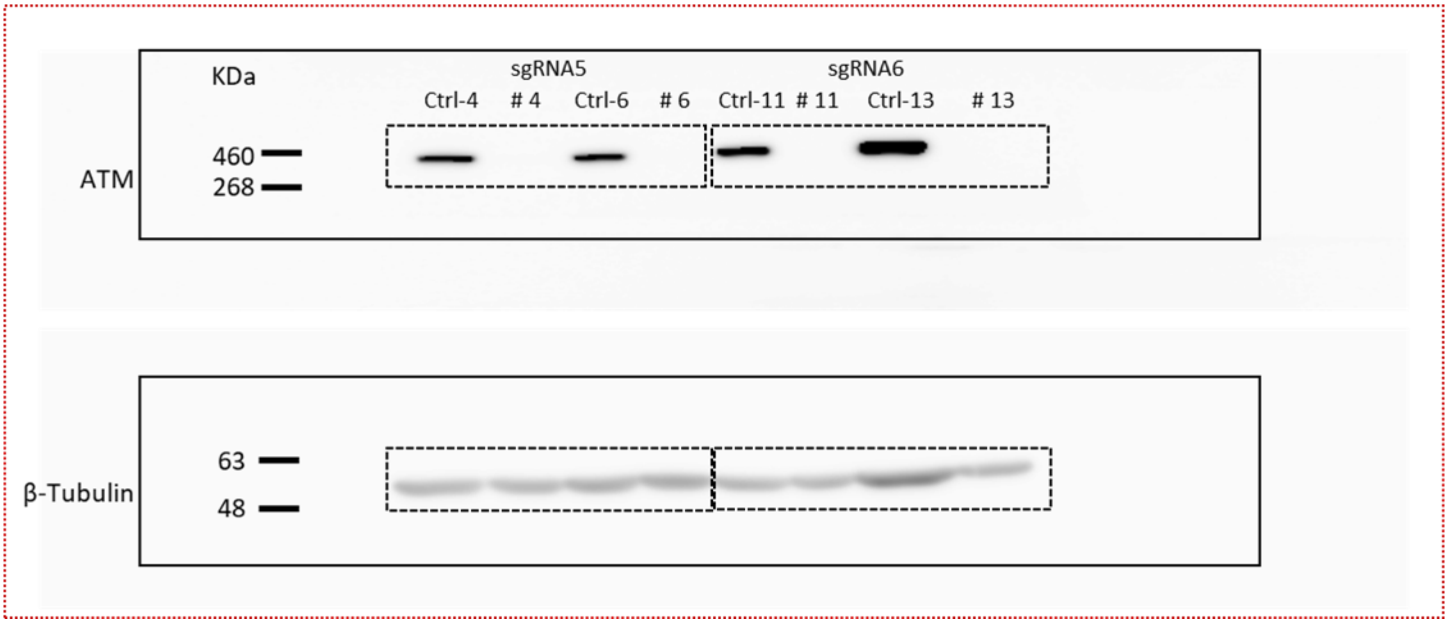

Full images of western blots shown in Figure 2A. Original full-length membrane was divided in to several parts to hybridization with different antibodies. Black block line indicates the edges of the membrane after cut. Black dotted lines indicate the cropping locations.

Full-length blots of Figure 3

Figure 3A

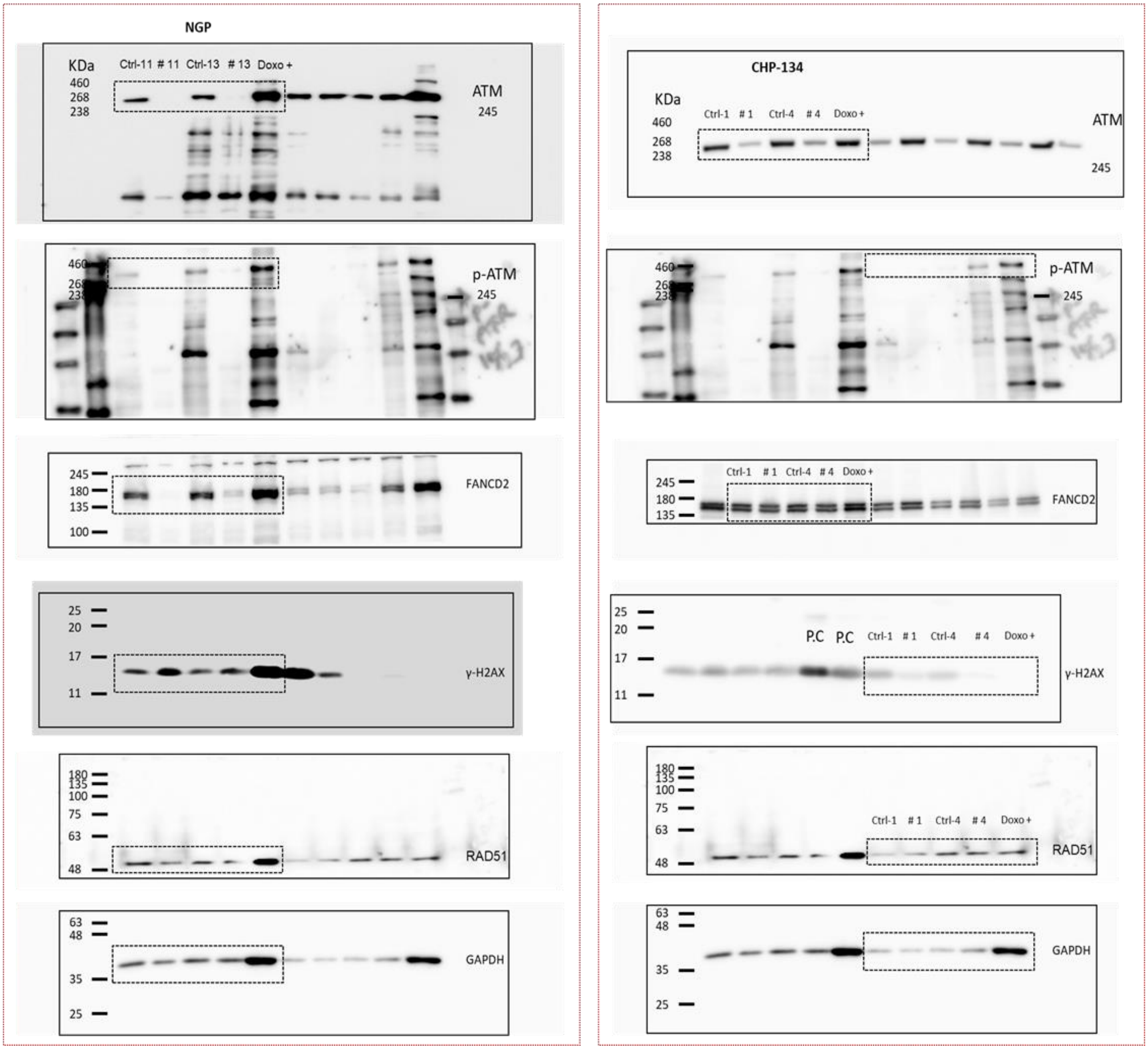

Full images of western blots shown in Figure 3A. Original full-length membrane was divided in to several parts to hybridization with different antibodies. Black block line indicates the edges of the membrane after cut. Black dotted lines indicate the cropping locations.

Full-length blots of Figure 4

Figure 4A

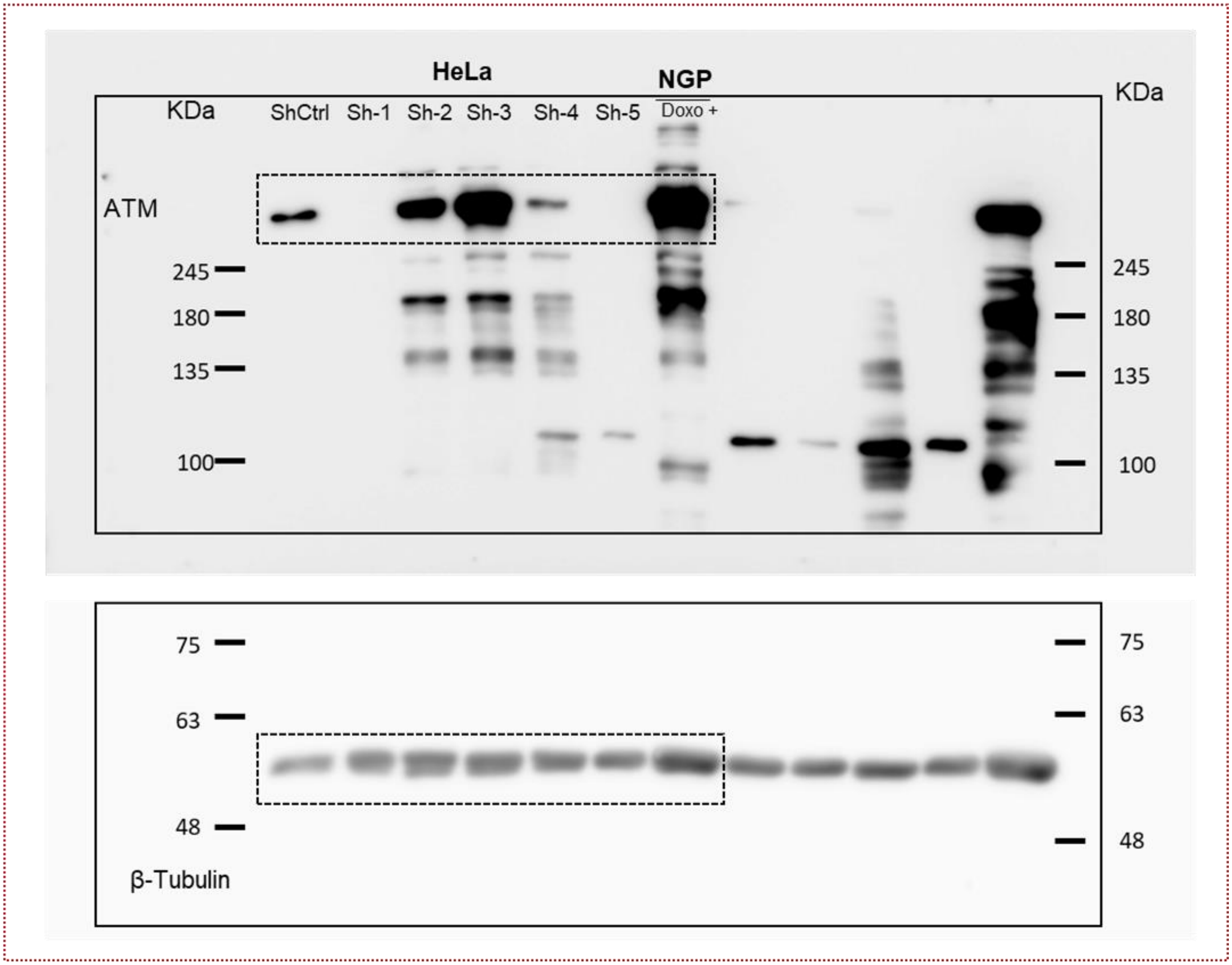

Full images of western blots shown in Figure 4A. Original full-length membrane was divided in to two parts. Black block line indicates the edges of the membrane after cut. Black dotted lines indicate the cropping locations.

Figure 4B

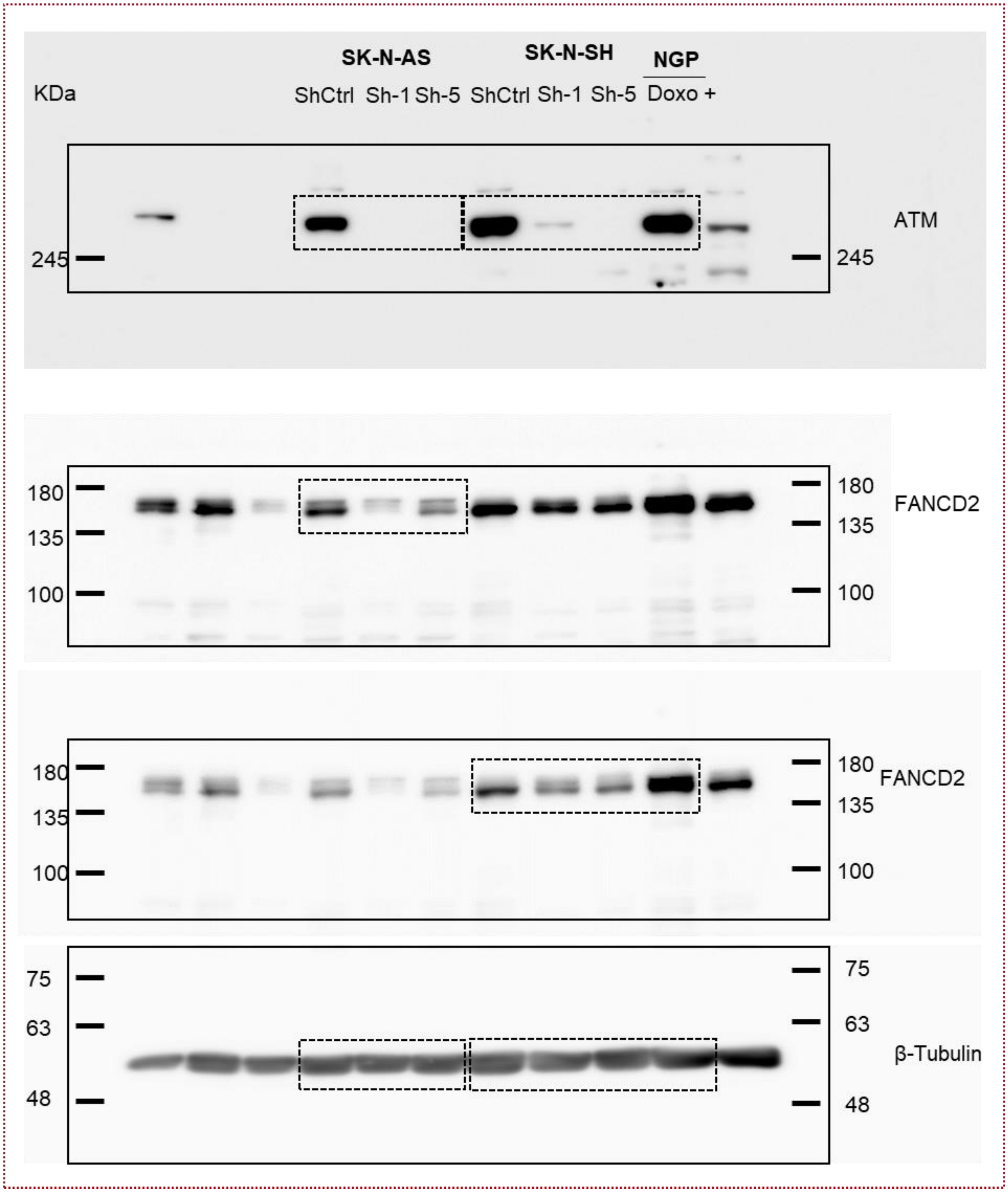

Full images of western blots shown in Figure 4B. Original full-length membrane was divided in to several parts to hybridization with different antibodies. Black block line indicates the edges of the membrane after cut. Black dotted lines indicate the cropping locations used for images shown in Figure 4B.

Full-length blots of Figure 5

Figure 5A

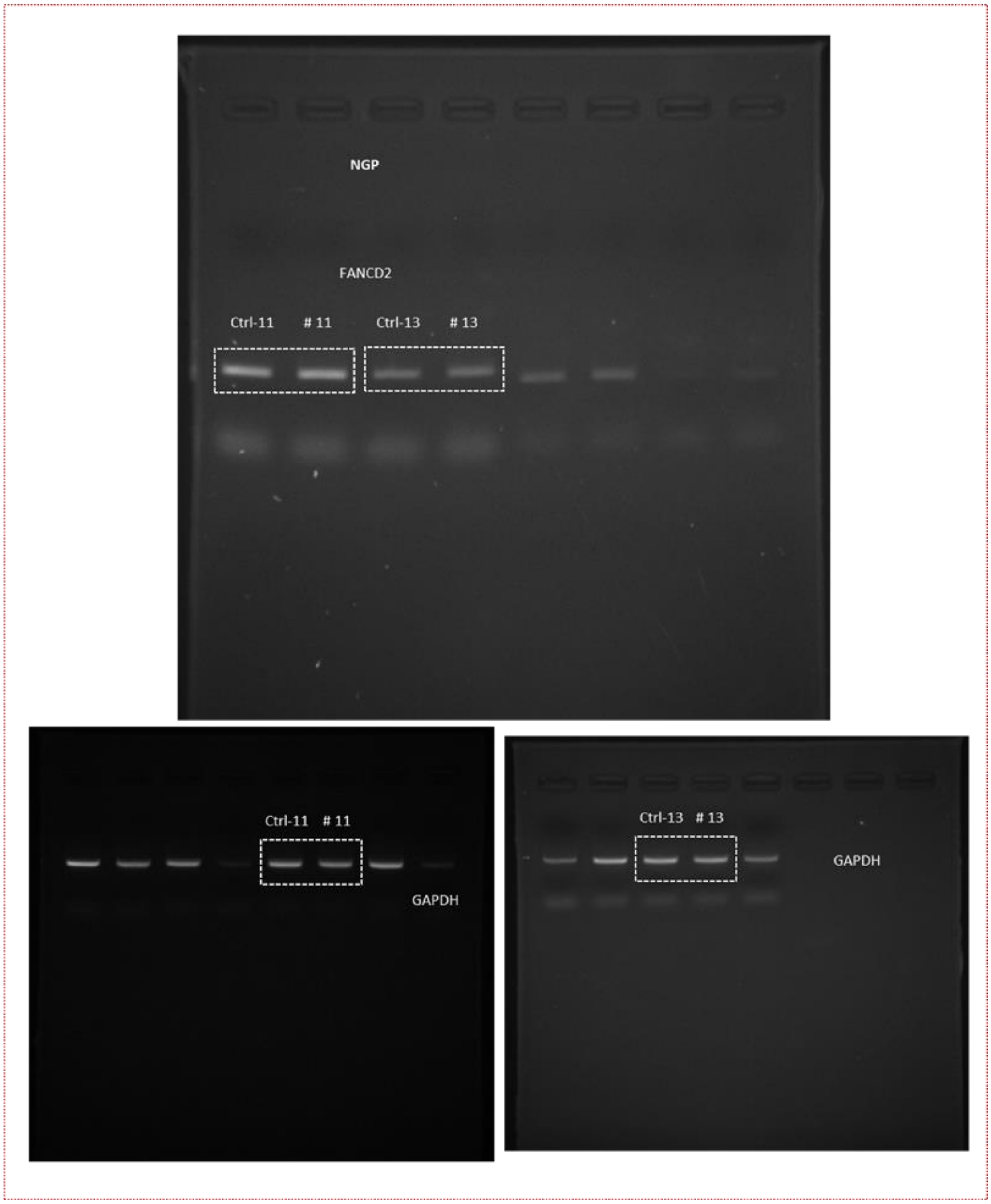

White dotted lines indicate the cropping locations used for images shown in Figure 5A.

Figure 5B

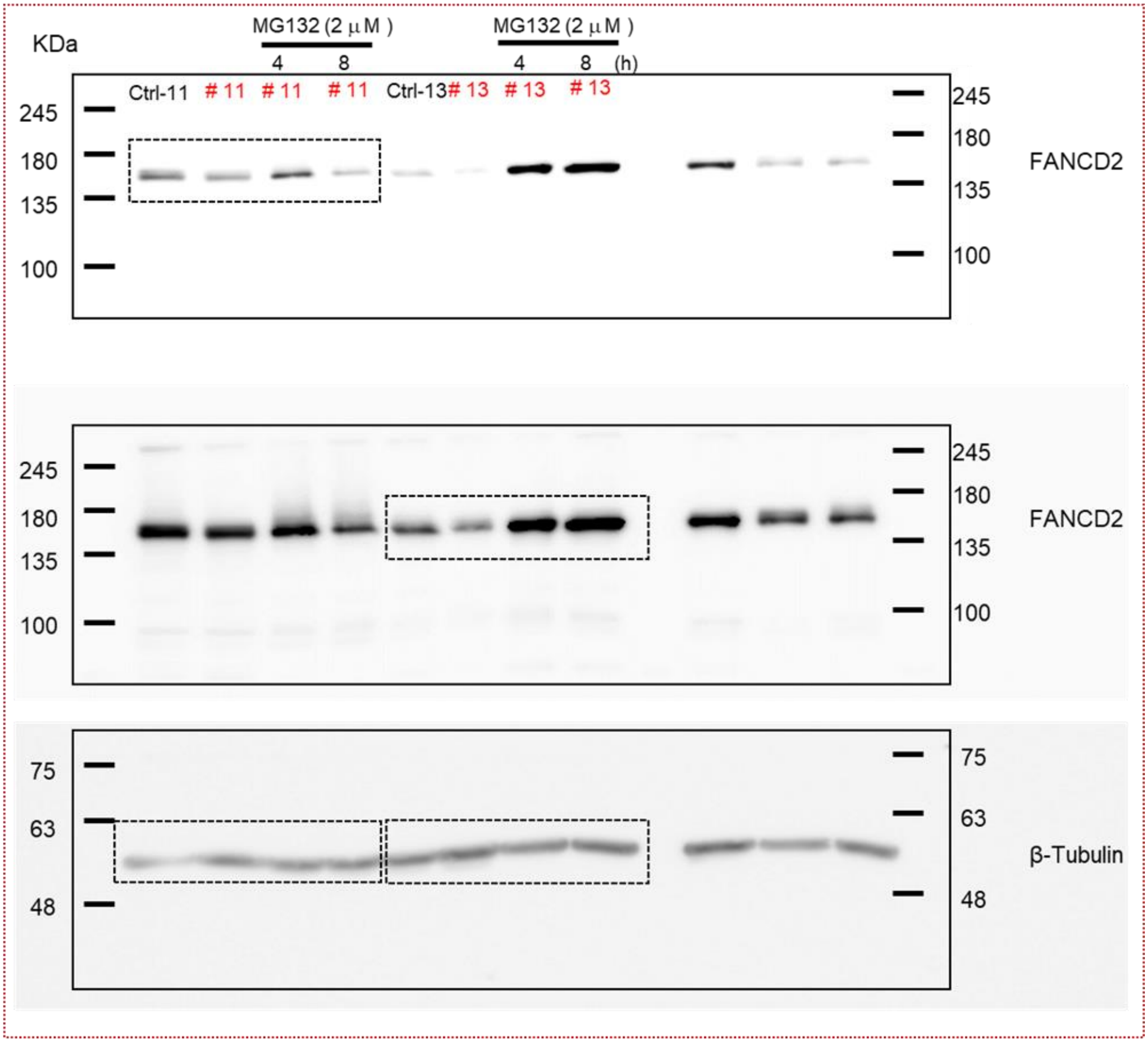

Full images of western blots shown in Figure 5B. Original full-length membrane was divided in to two parts. Black block line indicates the edges of the membrane after cut. Black dotted lines indicate the cropping locations used for images shown in Figure 5B.

Full-length blots of Figure 6

Fig. 6A

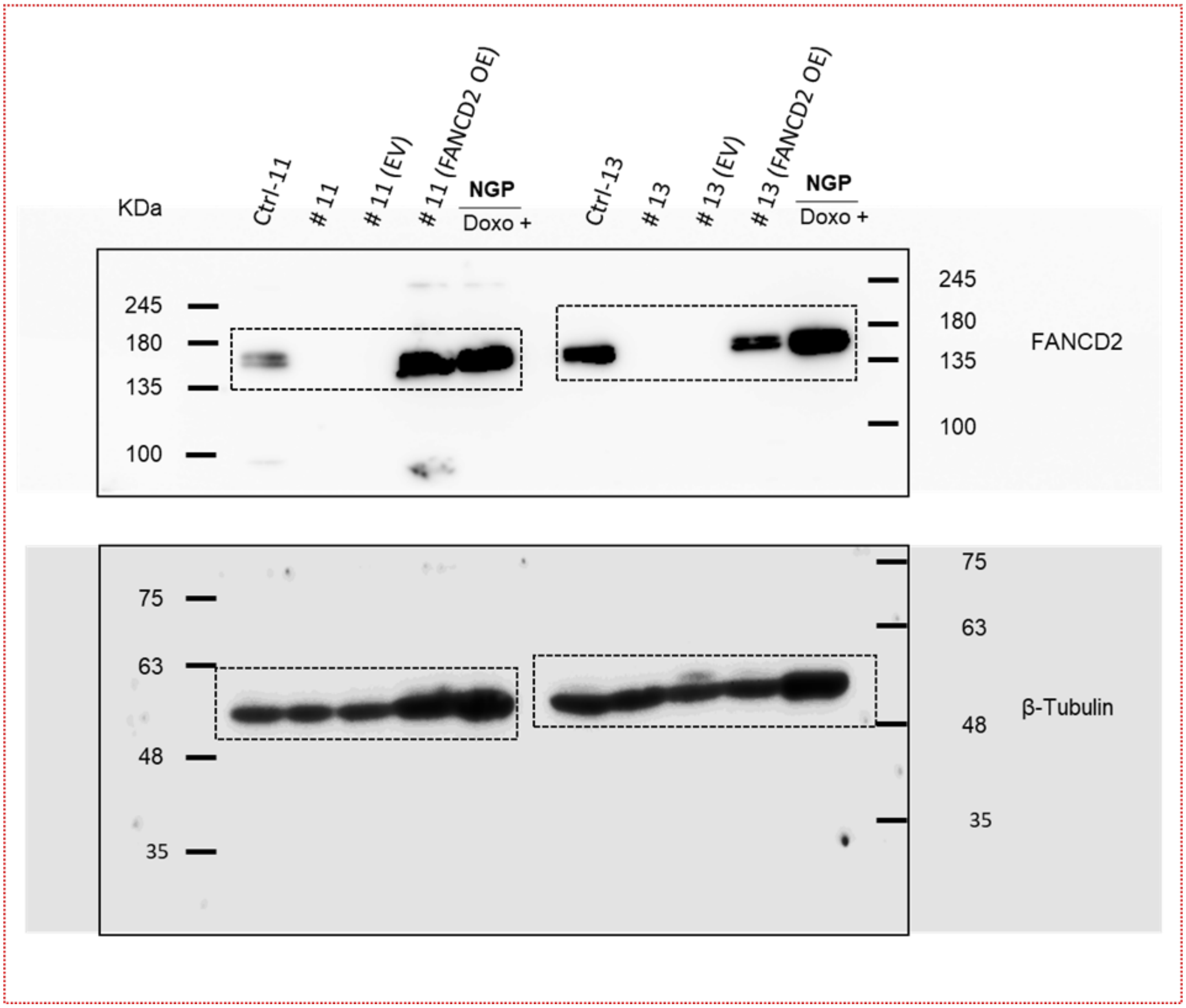

Full images of western blots shown in Figure 6A. Original full-length membrane was divided in to two parts. Black block line indicates the edges of the membrane after cut. Black dotted lines indicate the cropping locations used for images shown in Figure 6A.

Full-length blots of Supplementary Figure 2

Supplementary Figure 2B

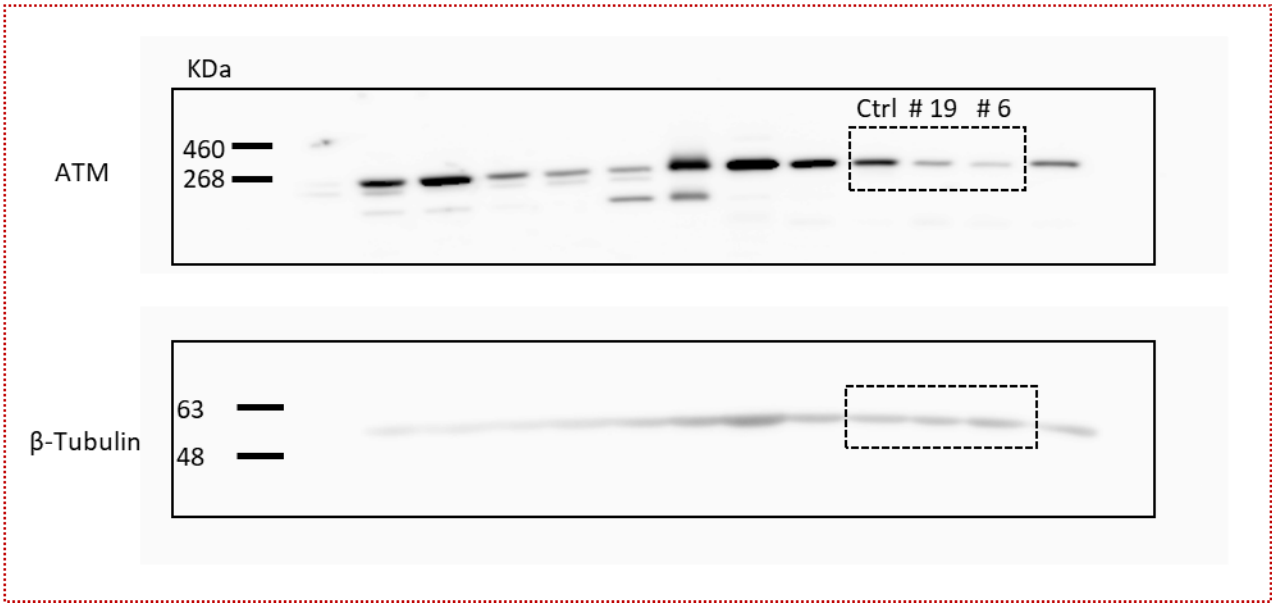

Full images of western blots shown in Supplementary Figure 2B. Original full-length membrane was divided in to two parts. Black block line indicates the edges of the membrane after cut. Black dotted lines indicate the cropping locations.

Full-length blots of Supplementary Figure 3

ATM/Chk2/p53 pathway

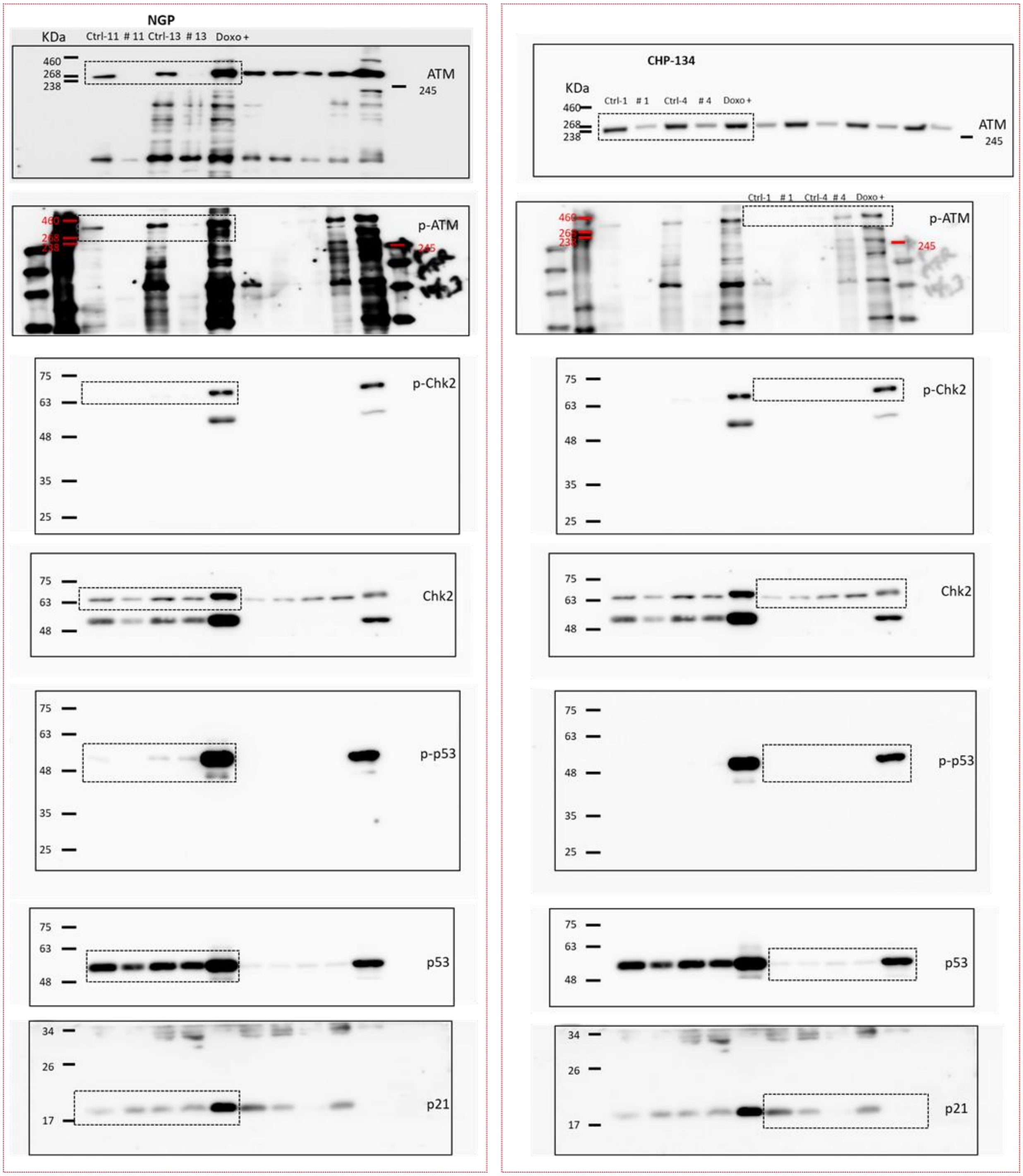

Full images of western blots shown in Supplementary Figure 3 (ATM/Chk2/p53 pathway). Original full-length membrane was divided in to several parts to hybridization with different antibodies. Black block line indicates the edges of the membrane after cut. Black dotted lines indicate the cropping locations.

Different exposure images of p-ATM in Supplementary Figure 3 (ATM/Chk2/p53 pathway)

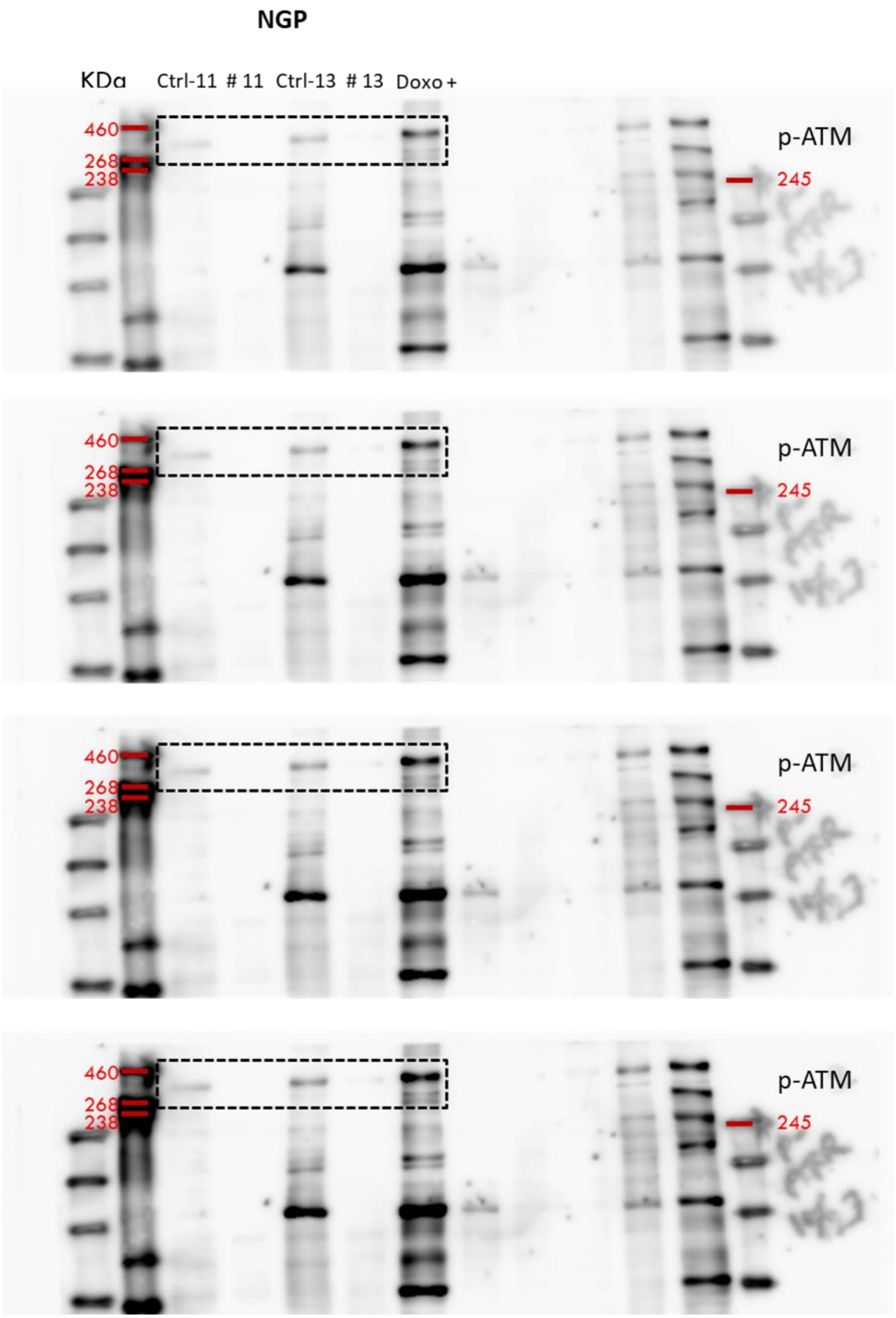

Supplementary Figure 3

ATR/Chk1 pathway

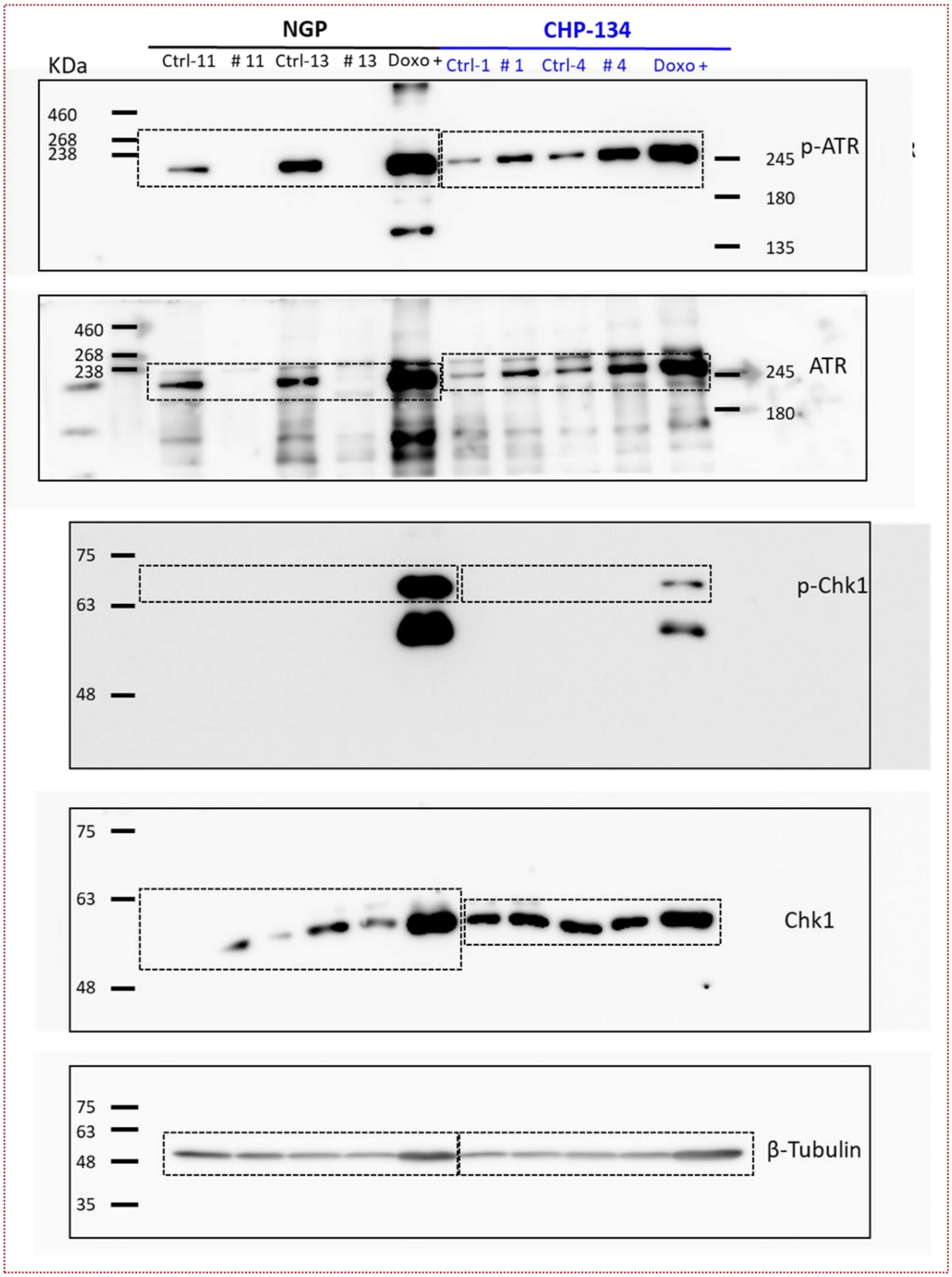

Full images of western blots shown in Supplementary Figure 3 (ATR/Chk1 pathway). Original full-length membrane was divided in to several parts to hybridization with different antibodies. Black block line indicates the edges of the membrane after cut. Black dotted lines indicate the cropping locations.
